# Supplementary material for: A Palette of Minimally Tagged Sucrose Analogues for Real‐Time Raman Imaging of Intracellular Plant Metabolism
Source: Angew Chem Weinheim Bergstr Ger. 2021 Feb 26;133(14):7715–20. doi: 10.1002/ange.202016802 (PMC10946860; doi:10.1002/ange.202016802)
Supplement: Supplementary file 1 — Supplementary [file ANGE-133-7715-s002.pdf]

Angewandte Chemie
Eine Zeitschrift der Gesellschaft Deutscher Chemiker

Supporting Information

A Palette of Minimally Tagged Sucrose Analogues for Real-Time Raman Imaging of Intracellular Plant Metabolism

*Fabio de Moliner, Kirsten Knox, Doireann Gordon, Martin Lee, William J. Tipping, Ailsa Geddis, Anke Reinders, John M. Ward, Karl Oparka, and Marc Vendrell**

ange_202016802_sm_miscellaneous_information.pdf
ange_202016802_sm_MovieS1.avi
ange_202016802_sm_MovieS2.avi

Electronic Supporting Information

Table of Contents

Supplementary Methods (Chemistry)

^{1}H and ^{13}C NMR spectra

Supplementary Methods (Spectroscopy/Biology/Imaging)

Supplementary Figures

Supplementary Tables

Supplementary References

1. Supplementary Methods (Chemistry)

General materials for chemical synthesis

Commercially available reagents were used without further purification. Thin-layer chromatography was conducted on Merck silica gel 60 F254 sheets and visualized by UV (254 and 365 nm). Silica gel (particle size 35−70 μm) was used for column chromatography. ^{1}H and ^{13}C spectra were recorded in a Bruker Avance 500 spectrometer (at 500 and 126 MHz, respectively). Data for ^{1}H NMR spectra are reported as chemical shift δ (ppm), multiplicity, coupling constant (Hz), and integration. Data for ^{13}C NMR spectra are reported as chemical shifts relative to the solvent peak. HPLC−MS analysis was performed on a Waters Alliance 2695 separation module connected to a Waters PDA2996 photo-diode array detector and a ZQ Micromass mass spectrometer (ESI-MS) with a Phenomenex column (C_{18}, 5 μm, 4.6 × 150 mm). Reverse phase chromatographic purification of the fully deprotected sucrose derivatives was conducted on Acros C18 reverse phase silica gel, ~17%C, approx. 0.7 mmol/g, particle size 48−65 μm. Fractions containing the products were identified by reverse phase thin-layer chromatography on Merck reverse phase RP-18 F254 sheets upon staining with phosphomolybdic acid and/or ninhydrin.

Chemical synthesis

1'-O-trifluoromethanesulfonyl-2,3,4,6,3',4',6'-hepta-O-benzoylsucrose (2)[1]

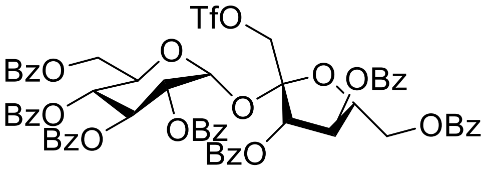

2,3,4,6,3',4',6'-Hepta-O-benzoylsucrose **1** (800 mg, 0.75 mmol, 1 eq) and 2,6-lutidine (157 μL, 1.34 mmol, 1.8 eq) were dissolved in dry DCM (16 mL) under a N_2 atmosphere and cooled to -78 °C. Trifluoromethanesulphonic anhydride (212 μL, 1.27 mmol, 1.7 eq) was added via a syringe and the reaction was stirred under N_2 at -78 °C for 1 h, upon which it was found complete (TLC). It was then quenched by adding H_2O (25 mL) and extracted with DCM (3 × 25 mL). Combined organics were washed with brine (50 mL), dried over $MgSO_4$ and concentrated under reduced pressure. The crude was purified by column chromatography (Hex:EtOAc, 7:3) to give **2** as a white solid (797 mg, 88% yield).

^{1}H NMR (500 MHz, $CDCl_3$) δ 8.28 – 8.21 (m, 2H), 8.08 – 7.99 (m, 6H), 7.95 (dd, *J* = 8.3, 1.2 Hz, 2H), 7.82 (dd, *J* = 8.3, 1.2 Hz, 2H), 7.69 (dd, *J* = 8.3, 1.2 Hz, 2H), 7.64 – 7.57 (m, 2H), 7.55 – 7.28 (m, 19H), 6.11 (dd, *J* = 11.3, 5.0 Hz, 2H), 5.94 (t, *J* = 10.0 Hz, 1H), 5.80 (d, *J* = 6.5 Hz, 1H), 5.71 (t, *J* = 9.8 Hz, 1H), 5.36 (dd, *J* = 10.4, 3.7 Hz, 1H), 4.87 (d, *J* = 10.5 Hz, 1H), 4.73 – 4.60 (m, 3H), 4.55 – 4.47 (m, 2H), 4.43 (d, *J* = 10.5 Hz, 1H), 4.31 (dd, *J* = 12.4, 3.0 Hz, 1H).

^{13}C NMR (125 MHz, $CDCl_3$) δ 166.0, 165.7, 165.6, 165.5, 165.3, 164.8, 134.0, 133.8, 133.6, 133.3, 133.1, 133.0, 130.2, 130.0, 129.84, 129.80, 129.77, 129.66, 129.4, 129.0, 128.9, 128.7, 128.6, 128.5, 128.34, 128.26, 102.6, 90.3, 79.3, 77.5, 74.8, 74.6, 70.9, 69.9, 69.4, 68.6, 63.4, 62.2.

MS (m/z, ESI): calcd for $C_{62}H_{50}F_3O_{20}S^+$ $[M+H]^+$: 1204.1, found: 1204.3.

1'-Azido-2,3,4,6,3',4',6'-hepta-O-benzoylsucrose (3)

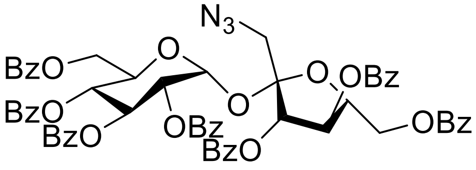

1'-O-trifluoromethanesulfonyl-2,3,4,6,3',4',6'-hepta-O-benzoylsucrose **2** (250 mg, 0.21 mmol, 1 eq) was dissolved in DMF (2 mL). Sodium azide (20 mg, 0.31 mmol, 1.5 eq) was added and reaction was stirred overnight at r.t. It was then diluted with EtOAc (50 mL), washed with H_2O (2 × 25 mL) and brine (50 mL). Organic phase was dried over $MgSO_4$, filtered and concentrated to dryness under reduced pressure to give **3** as a white solid (136 mg, 59% yield).

^{1}H NMR (500 MHz, $CDCl_3$) δ 8.23 (d, *J* = 7.6 Hz, 2H), 8.08 – 8.00 (m, 6H), 7.96 (d, *J* = 7.6 Hz, 2H), 7.84 (d, *J* = 7.6 Hz, 2H), 7.77 (d, *J* = 7.6 Hz, 2H), 7.60 (t, *J* = 7.4 Hz, 2H), 7.54 – 7.27 (m, 19H), 6.12 (d, *J* = 3.5 Hz, 1H), 6.11 – 6.01 (m, 2H), 5.95 (dd, *J* = 6.3, 1.2 Hz, 1H), 5.75 (t, *J* = 9.9 Hz, 1H), 5.43 – 5.33 (m, 1H), 4.72 (d, *J* = 5.6 Hz, 2H), 4.64 (dd, *J* = 11.4, 4.7 Hz, 2H), 4.57 (dd, *J* = 12.5, 2.6 Hz, 1H), 4.39 (dd, *J* = 12.5, 2.9 Hz, 1H), 3.63 (d, *J* = 12.8 Hz, 1H), 3.49 (d, *J* = 12.9 Hz, 1H).

^{13}C NMR (125 MHz, $CDCl_3$) δ 166.02, 166.01, 165.61, 165.60, 165.6, 165.3, 165.0, 133.8, 133.7, 133.5, 133.3, 133.08, 133.07, 132.96, 130.2, 129.93, 129.89, 129.82, 129.80, 129.74, 129.66, 129.55, 129.1, 128.81, 128.79, 128.76, 128.74, 128.6, 128.5, 128.32, 128.30, 128.27, 128.26, 105.4, 90.3, 78.9, 77.2, 75.6, 71.2, 70.1, 69.3, 68.8, 64.0, 62.3, 55.1.

MS (m/z, ESI): calcd for $C_{61}H_{49}N_3NaO_{17}^+$ $[M+Na]^+$: 1118.3, found: 1118.6.

1'-Deoxy-1'-azidosucrose (4)

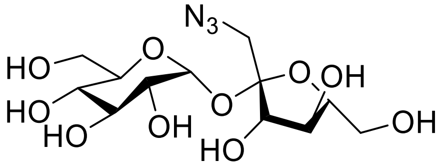

1'-Azido-2,3,4,6,3',4',6'-hepta-O-benzoylsucrose **3** (131 mg, 0.12 mmol, 1 eq) was dissolved in MeOH (3 mL), and K_2CO_3 (16 mg, 0.12 mmol, 1 eq) was added. Reaction was then heated to reflux for 5 min, upon which it was found complete (TLC, HPLC-MS). It was cooled to r.t., filtered through a Celite pad rinsing with further MeOH (40 mL) and the filtrate was concentrated to dryness under reduced pressure to give the crude product, which was purified by reverse phase column chromatography (H_2O:MeCN, 97:3) to give **4** as a colorless solid (50 mg, quantitative yield).

^{1}H NMR (500 MHz, D_2O) δ 5.38 (d, *J* = 3.7 Hz, 1H), 4.17 (dd, *J* = 8.8, 0.9 Hz, 1H), 3.99 (td, *J* = 8.7, 0.9 Hz, 1H), 3.93 – 3.83 (m, 1H), 3.81 – 3.71 (m, 5H), 3.71 – 3.61 (m, 2H), 3.52 – 3.48 (m, 1H), 3.45 – 3.38 (m, 1H), 3.36 – 3.29 (m, 1H).

^{13}C NMR (125 MHz, D_2O) δ 103.7, 92.4, 81.2, 76.6, 73.5, 72.53, 72.49, 71.0, 69.2, 62.1, 60.1, 52.2.

HRMS (m/z, ESI): calcd for $C_{12}H_{21}N_3NaO_{10}^+$ [M+Na]$^+$: 390.1119, found: 390.1108.

1'-Propargylamino-2,3,4,6,3',4',6'-hepta-O-benzoylsucrose (5)

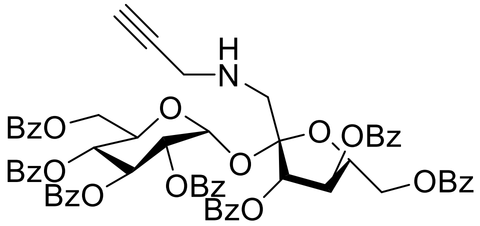

1'-O-trifluoromethanesulfonyl-2,3,4,6,3',4',6'-hepta-O-benzoylsucrose **2** (797 mg, 0.66 mmol, 1 eq) was dissolved in MeCN (8 mL). Propargylamine (212 μL, 3.31 mmol, 5 eq) was added and reaction was stirred at 50 °C for 3 days. It was then cooled down to r.t. and concentrated to dryness under reduced pressure to afford the crude product, which was purified by column chromatography (Hex:EtOAc, 67:33) to give **5** as a white solid (544 mg, 68% yield).

^{1}H NMR (500 MHz, $CDCl_3$) δ 8.26 (dd, *J* = 8.3, 1.3 Hz, 2H), 8.07 – 8.00 (m, 6H), 7.96 (dd, *J* = 8.3, 1.3 Hz, 2H), 7.89 – 7.81 (m, 4H), 7.64 – 7.30 (m, 21H), 6.21 – 6.14 (m, 2H), 6.09 (d, *J* = 3.6 Hz, 1H), 6.03 (t, *J* = 6.8 Hz, 1H), 5.79 (t, *J* = 9.9 Hz, 1H), 5.43 (dd, *J* = 10.4, 3.6 Hz, 1H), 4.79 – 4.68 (m, 4H), 4.60 (t, *J* = 6.4 Hz, 2H), 4.52 (dd, *J* = 12.5, 3.3 Hz, 2H), 3.24 (dd, *J* = 17.2, 2.4 Hz, 1H), 3.15 (dd, *J* = 17.2, 2.4 Hz, 1H), 3.00 (d, *J* = 13.0 Hz, 1H), 2.94 (d, *J* = 13.0 Hz, 1H), 1.91 (t, *J* = 2.3 Hz, 1H), 1.60 (s, 1H).

^{13}C NMR (125 MHz, $CDCl_3$) δ 166.1, 166.0, 165.65, 165.59, 165.57, 165.5, 165.1, 133.50, 133.45, 133.35, 133.30, 133.02, 132.99, 132.9, 130.2, 129.95, 129.94, 129.83, 129.80, 129.66, 129.63, 129.3, 129.2, 129.1, 128.96, 128.92, 128.8, 128.5, 128.4, 128.29, 128.28, 128.25, 106.3, 90.0, 81.5, 78.7, 76.2, 71.4, 71.3, 70.3, 69.0, 64.7, 62.5, 52.1, 38.1.

MS (m/z, ESI): calcd for $C_{64}H_{54}NO_{17}^+$ $[M+H]^+$: 1108.3, found: 1108.5.

1'-Deoxy-1'-propargylaminosucrose (6)

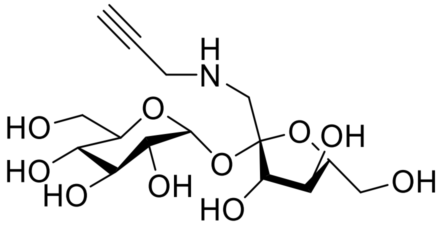

1'-Propargylamino-2,3,4,6,3',4',6'-hepta-O-benzoylsucrose **5** (544 mg, 0.49 mmol, 1 eq) was dissolved in MeOH (40 mL), and K_2CO_3 (68 mg, 0.49 mmol, 1 eq) was added. Reaction was then stirred at r.t. for 4 h, upon which it was found complete (TLC, HPLC-MS). It was filtered through a Celite pad rinsing with further MeOH (40 mL) and the filtrate was concentrated to dryness under reduced pressure to give the crude product, which was purified by reverse phase column chromatography (H_2O:MeCN, 97:3) to give **6** as a colorless solid (186 mg, quantitative yield).

^{1}H NMR (500 MHz, D_2O) δ 5.39 (d, *J* = 3.8 Hz, 1H), 4.08 (d, *J* = 8.6 Hz, 1H), 3.97 (t, *J* = 8.5 Hz, 1H), 3.86 – 3.81 (m, 1H), 3.80 – 3.68 (m, 7H), 3.49 (dd, *J* = 10.0, 3.8 Hz, 1H), 3.44 – 3.34 (m, 3H), 2.93 (q, *J* = 12.9 Hz, 2H).

^{13}C NMR (125 MHz, D_2O) δ 105.2, 103.4, 92.1, 81.3, 78.4, 74.1, 72.8, 72.5, 71.2, 69.3, 62.4, 60.1, 51.1, 48.9, 37.1.

HRMS (m/z, ESI): calcd for $C_{15}H_{26}NO_{10}^+$ $[M+H]^+$: 380.1551, found: 380.1543.

2,3,4,6,3',4',6'-Hepta-O-benzoylsucrose aldoxime (8)

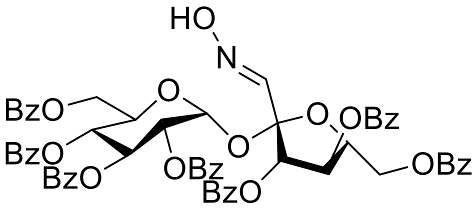

2,3,4,6,3',4',6'-Hepta-O-benzoylsucrose **1** (500 mg, 0.46 mmol, 1 eq) was dissolved in DCM (15 mL) under a N_2 atmosphere, then 0.3 M DCM solution of Dess-Martin periodinane (2.4 mL, 0.73 mmol, 1.6 eq) was added via a syringe and reaction was stirred at r.t. for 1 h under N_2. Upon completion (TLC, HPLC-MS) it was diluted with Et_2O (40 mL), and $Na_2S_2O_{3\ sat}$ (25 mL) and $NaHCO_{3\ sat}$ (25 mL) were added. The mixture was then extracted with further Et_2O (3 × 25 mL) and combined organics were dried over $MgSO_4$, filtered and concentrated to dryness under reduced pressure to afford crude 2,3,4,6,3',4',6'-hepta-O-benzoylsucrose aldehyde **7** as a thick colorless oil that was immediately dissolved in MeOH (20 mL). $NH_2OH \cdot HCl$ (64 mg, 0.92 mmol, 2 eq) and AcONa (45 mg, 0.55 mmol, 1.2 eq) were added and reaction was stirred for 2 h at 40 °C. Upon completion (HPLC-MS) it was diluted with H_2O (100 mL) and extracted with DCM (3 × 50 mL). Combined organics were washed with brine (2 × 50 mL), dried over $MgSO_4$, filtered and concentrated to dryness under reduced pressure to afford the crude product, which was purified by column chromatography (Hex/EtOAc, 6:4) to give **8** as a thick colorless oil (267 mg, 54 % yield).

^{1}H NMR (500 MHz, DMSO-d_6) δ 11.48 (s, 1H), 8.11 (d, J = 7.3 Hz, 2H), 8.01 – 7.91 (m, 6H), 7.75 – 7.34 (m, 25H), 7.23 (t, J = 7.8 Hz, 2H), 6.24 (d, J = 5.6 Hz, 1H), 6.05 (t, J = 10.0 Hz, 1H), 6.01 (d, J = 3.6 Hz, 1H), 5.96 (t, J = 5.5 Hz, 1H), 5.77 – 5.72 (m, 1H), 5.59 (dd, J = 10.3, 3.6 Hz, 1H), 4.83 – 4.72 (m, 4H), 4.51 – 4.42 (m, 2H).

^{13}C NMR (125 MHz, DMSO-d_6) δ 165.8, 165.8, 165.5, 165.5, 165.2, 165.1, 165.0, 145.2, 134.3, 134.3, 134.2, 134.1, 133.9, 133.8, 130.1, 130.0, 129.8, 129.7, 129.6, 129.5, 129.3, 129.2, 129.1, 129.0, 129.0, 128.9, 128.9, 128.8, 128.7, 104.3, 91.8, 78.8, 77.6, 77.1, 71.0, 70.7, 69.2, 69.0, 65.3, 62.9.

MS (m/z, ESI): calcd for $C_{61}H_{49}NNaO_{18}^{+}$ $[M+Na]^{+}$: 1107.0, found: 1107.6.

2,3,4,6,3',4',6'-Hepta-O-benzoylsucrose nitrile (9)

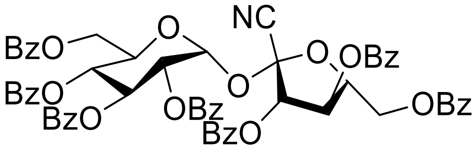

2,3,4,6,3',4',6'-Hepta-O-benzoylsucrose aldoxime **8** (208 mg, 0.19 mmol, 1 eq) was dissolved in DCM (10 mL), then CDI (37 mg, 0.23 mmol, 1.2 eq) was added and reaction was stirred at r.t. for 7 h. Solvent was then removed under reduced pressure and the crude product was purified by column chromatography (Hex/EtOAc, 6:4) to give **9** as a thick colorless oil (184 mg, 91 % yield).

^{1}H NMR (500 MHz, $CDCl_3$) δ 8.25 – 8.18 (m, 2H), 8.11 – 8.00 (m, 6H), 7.87 – 7.76 (m, 6H), 7.68 – 7.60 (m, 2H), 7.56 – 7.46 (m, 7H), 7.46 – 7.38 (m, 4H), 7.37 – 7.32 (m, 4H), 7.30 – 7.26 (m, 2H), 7.23 – 7.18 (m, 2H), 6.14 (d, *J* = 3.7 Hz, 1H), 6.10 (d, *J* = 3.3 Hz, 1H), 6.00 (t, *J* = 10.1 Hz, 1H), 5.87 (t, *J* = 3.6 Hz, 1H), 5.74 (t, *J* = 10.0 Hz, 1H), 5.49 (dd, *J* = 10.4, 3.7 Hz, 1H), 4.83 (dd, *J* = 11.6, 5.1 Hz, 1H), 4.78 – 4.67 (m, 2H), 4.61 – 4.65 (m, 2H), 4.44 – 4.35 (m, 1H).

^{13}C NMR (125 MHz, $CDCl_3$) δ 166.0, 165.9, 165.7, 165.2, 165.1, 165.0, 164.5, 134.0, 133.9, 133.4, 133.3, 133.3, 133.1, 133.0, 130.4, 130.1, 130.0, 129.9, 129.8, 129.7, 129.6, 129.2, 129.0, 128.9, 128.7, 128.5, 128.4, 128.3, 128.3, 128.2, 128.2, 128.0, 113.8, 99.0, 95.4, 81.9, 79.4, 75.6, 70.7, 70.0, 69.7, 68.7, 63.3, 62.2.

HRMS (m/z, ESI): calcd for $C_{61}H_{47}NNaO_{17}^+$ $[M+Na]^+$: 1088.2736, found: 1088.2712.

1'-Propionitrilamino-2,3,4,6,3',4',6'-hepta-O-benzoylsucrose (11)

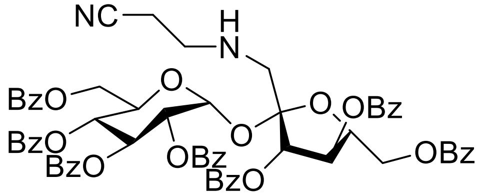

1'-O-trifluoromethanesulfonyl-2,3,4,6,3',4',6'-hepta-O-benzoylsucrose **2** (34 mg, 0.03 mmol, 1 eq) was dissolved in MeCN (1 mL). 2-Aminopropionitrile (11 μL, 0.14 mmol, 5 eq) was added and reaction was stirred at 50 °C for 2 days. It was then cooled down to r.t. and concentrated to dryness under reduced pressure to afford the crude product, which was purified by column chromatography (Hex:EtOAc, 7:3) to give **11** as a white amorphous solid (18 mg, 60% yield).

^{1}H NMR (500 MHz, $CDCl_3$) δ 8.23 (d, *J* = 7.1, 2H), 8.11 – 7.93 (m, 9H), 7.87 (dd, *J* = 8.3, 1.2 Hz, 2H), 7.82 (d, *J* = 7.1, 2H), 7.59 (dd, *J* = 8.8, 7.5 Hz, 2H), 7.54 – 7.33 (m, 18H), 6.18 – 6.06 (m, 3H), 6.04 (t, *J* = 6.9 Hz, 1H), 5.78 (t, *J* = 9.9 Hz, 1H), 5.35 (dd, *J* = 10.4, 3.5 Hz, 1H), 4.78 – 4.68 (m, 3H), 4.61 (dd, *J* = 12.6, 5.9 Hz, 2H), 4.49 – 4.45 (m, 1H), 4.15 (q, J = 7.1 Hz, 1H), 3.04 – 2.60 (m, 4H).

13C NMR (125 MHz, CDCl3) δ 166.04, 166.01, 165.9, 165.7, 165.5, 165.4, 165.0, 133.74, 133.68, 133.56, 133.3, 133.1, 133.03, 132.96, 130.2, 129.99, 129.95, 129.91, 129.82, 129.79, 129.75, 129.68, 129.58, 129.09, 128.89, 128.87, 128.84, 128.80, 128.7, 128.6, 128.5, 128.4, 128.32, 128.30, 128.28, 106.4, 105.8, 90.1, 71.6, 70.0, 69.2, 68.8, 64.4, 62.4, 60.4, 52.8, 45.1, 29.7, 21.1, 14.2.

HRMS (m/z, ESI): calcd for $C_{64}H_{55}N_2O_{17}{}^+$ [M+H]$^+$: 1123.3495, found: 1123.3500.

1'-Deoxy-1'-propionitrilaminosucrose (12)

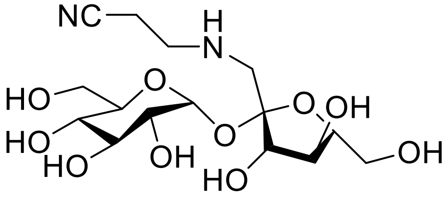

1'-Propionitrilo-2,3,4,6,3',4',6'-hepta-O-benzoylsucrose **11** (18 mg, 0.02 mmol, 1 eq) was dissolved in MeOH (2 mL), and K_2CO_3 (2.2 mg, 0.02 mmol, 1 eq) was added. Reaction was then stirred at r.t. for 5 h, upon which it was found complete (TLC, HPLC-MS). It was filtered through a Celite pad rinsing with further MeOH (40 mL) and the filtrate was concentrated to dryness under reduced pressure to give the crude product, which was purified by reverse phase column chromatography (H_2O:MeCN, 97:3) to give **12** as a white solid (5 mg, 94% yield).

^{1}H NMR (500 MHz, D_2O) δ 5.40 (d, *J* = 7.3, 3.9 Hz, 1H), 4.17 – 3.84 (m, 3H), 3.81 – 3.68 (m, 7H), 3.64 – 3.54 (m, 1H), 3.48 (ddd, *J* = 10.0, 3.8, 1.8 Hz, 1H), 3.41 (td, *J* = 9.4, 3.2 Hz, 1H), 3.31 – 3.20 (m, 1H), 2.93 – 2.84 (m, 2H), 2.76 – 2.59 (m, 2H).

13C NMR (125 MHz, D2O) δ 120.5, 120.3, 105.2, 103.7, 103.4, 92.2, 81.4, 81.0, 78.6, 76.4, 76.2, 74.05, 73.98, 73.4, 72.9, 72.6, 72.4, 72.3, 71.2, 71.1, 69.3, 69.2, 62.3, 61.3, 60.1, 51.7, 49.5, 44.1, 17.2, 16.3.

HRMS (m/z, ESI): calcd for $C_{15}H_{27}N_2O_{10}{}^+$ $[M+H]^+$: 395.1660, found: 395.1644.

^{1}H and ^{13}C NMR spectra

Compound **2**

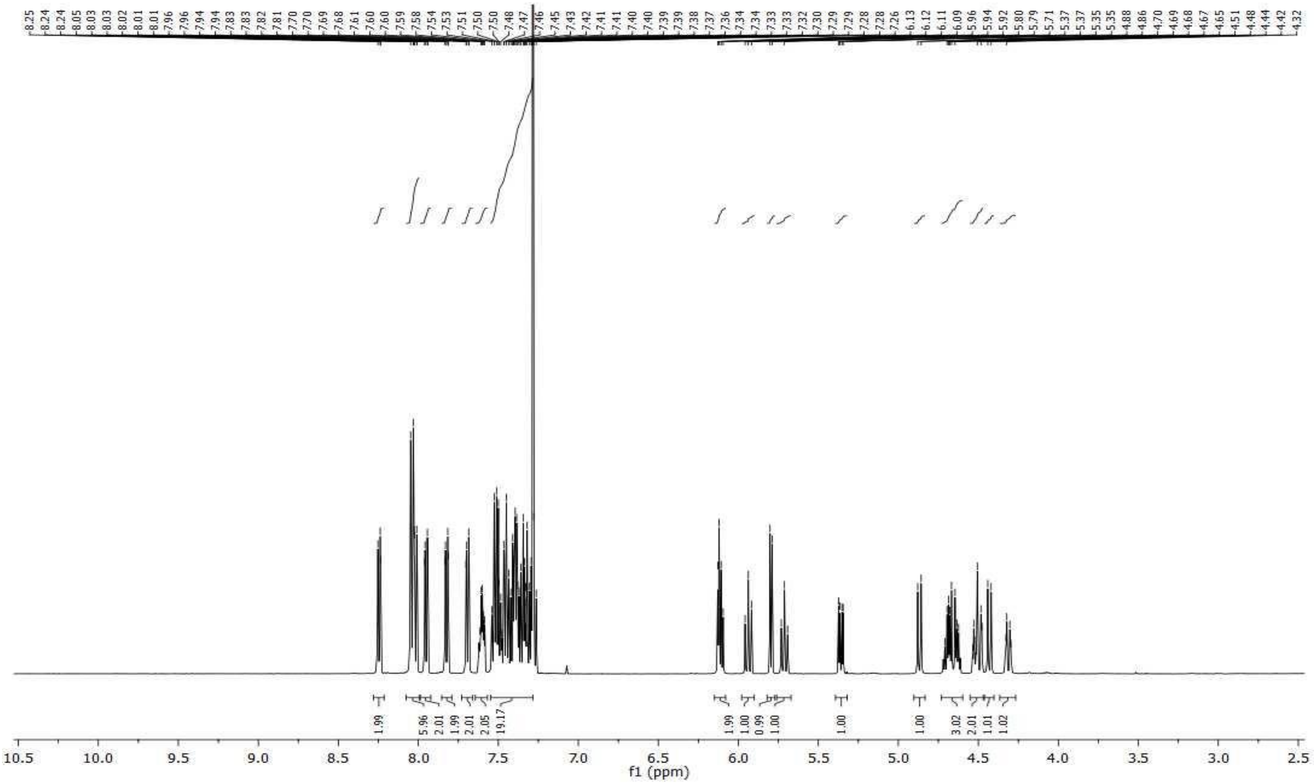

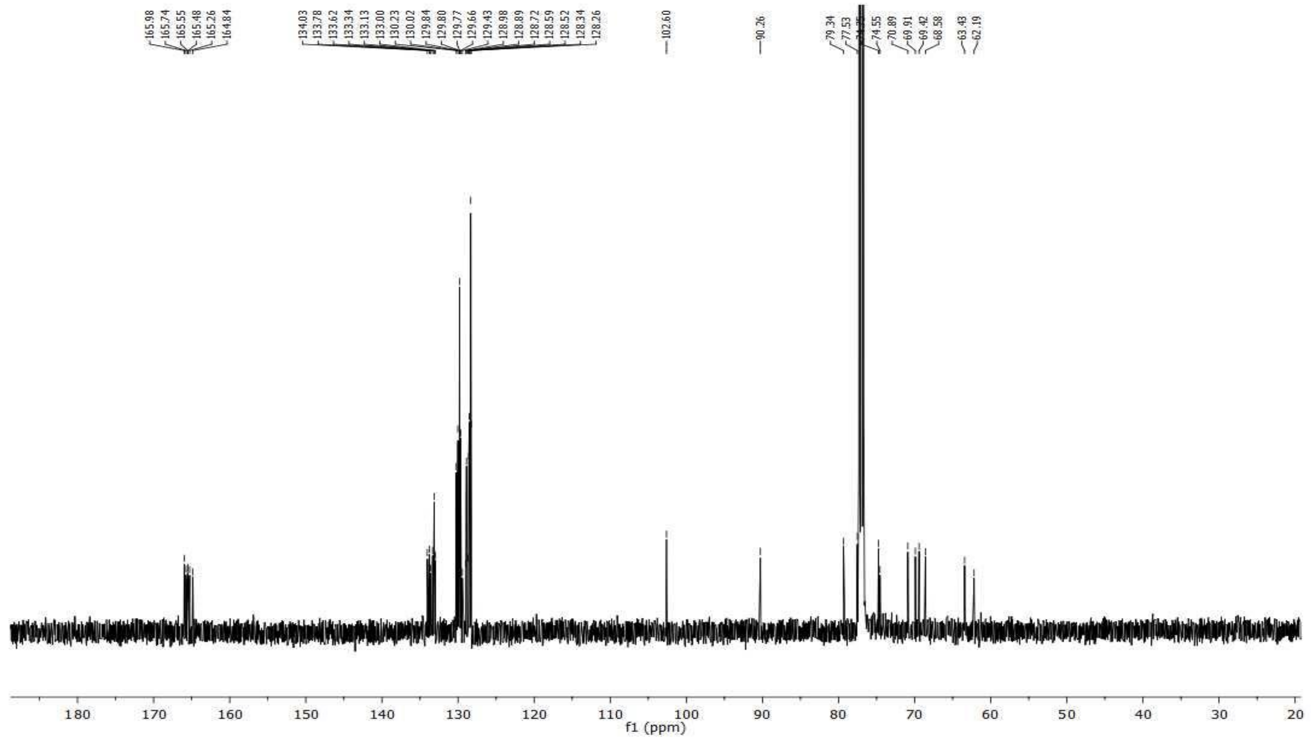

Compound **3**

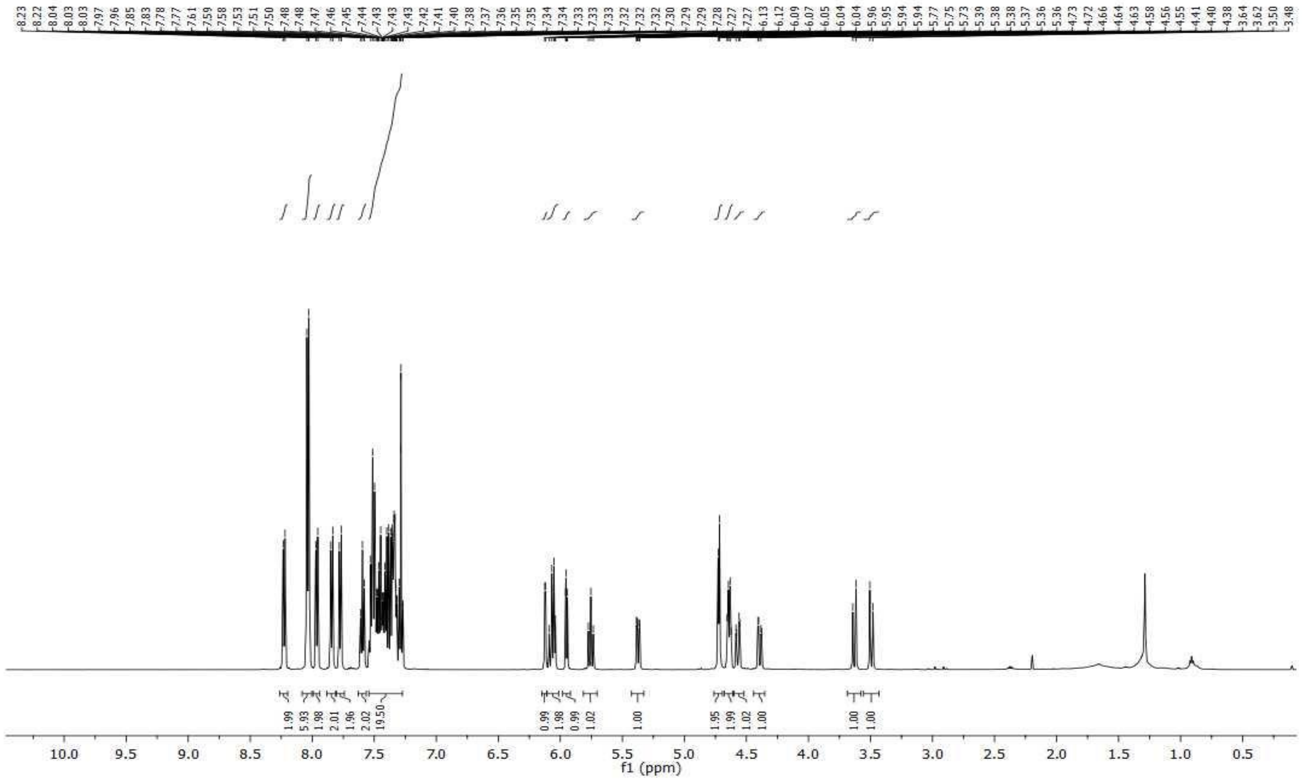

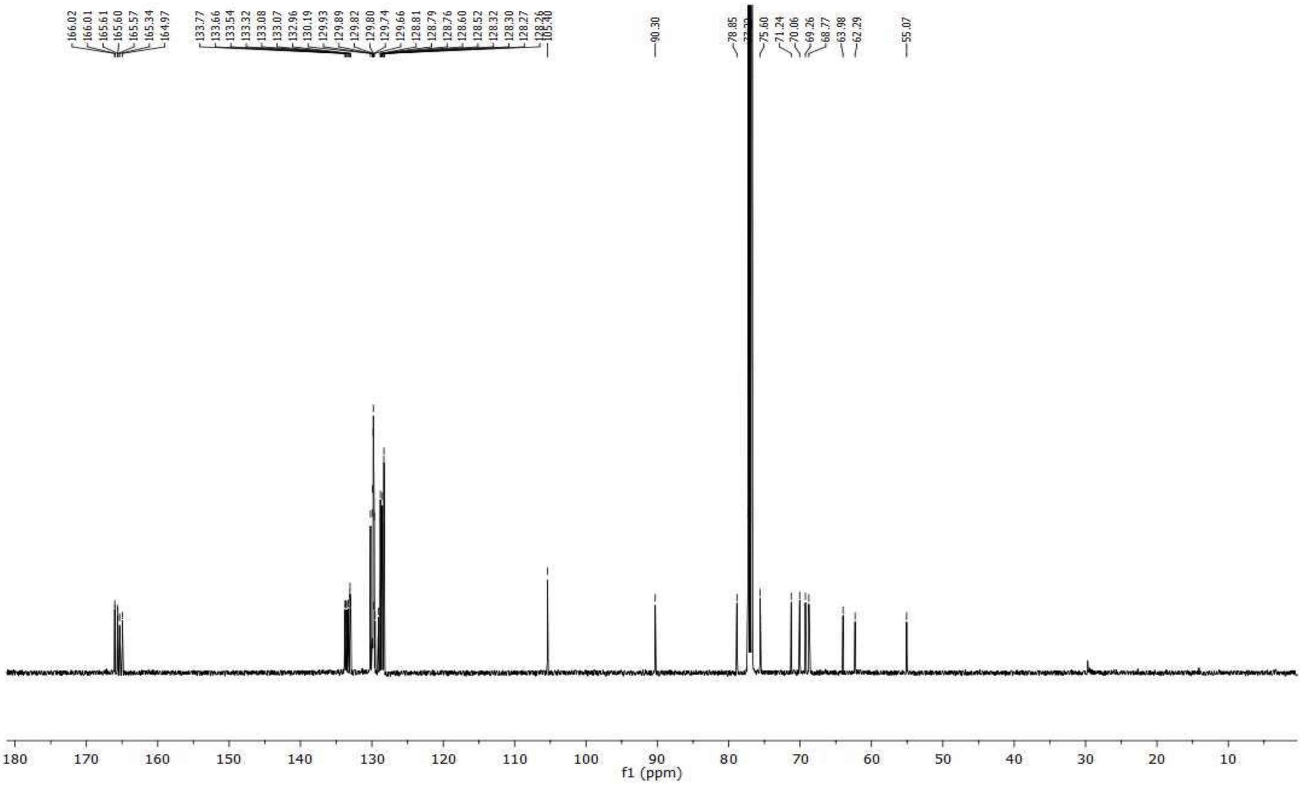

Compound **4**

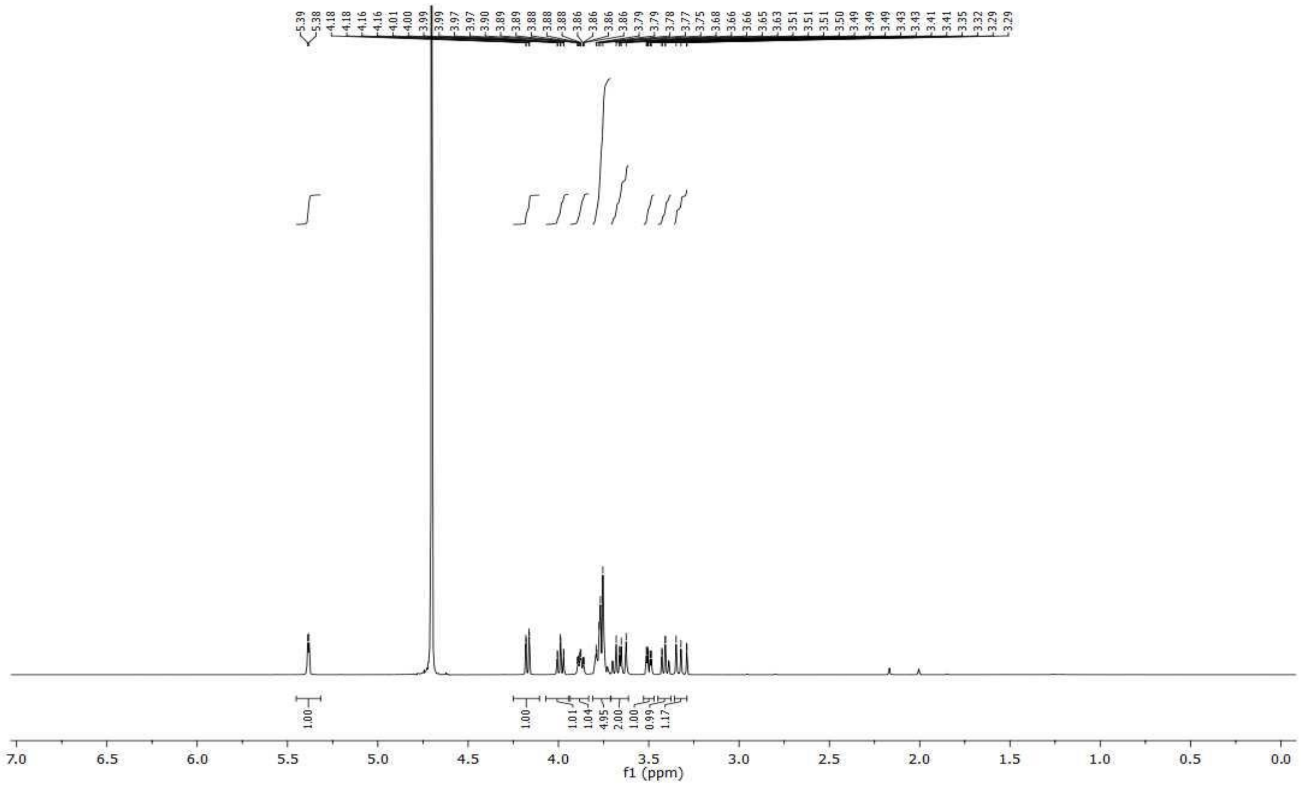

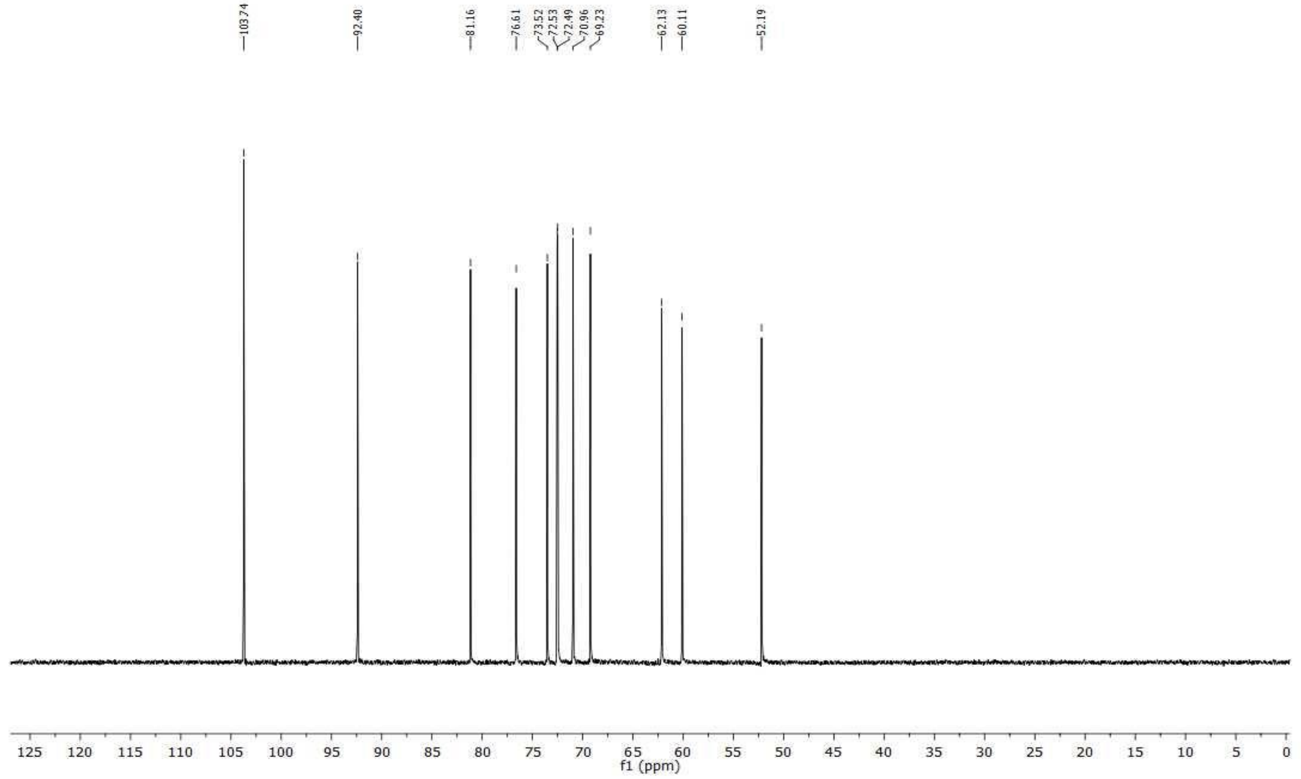

Compound **5**

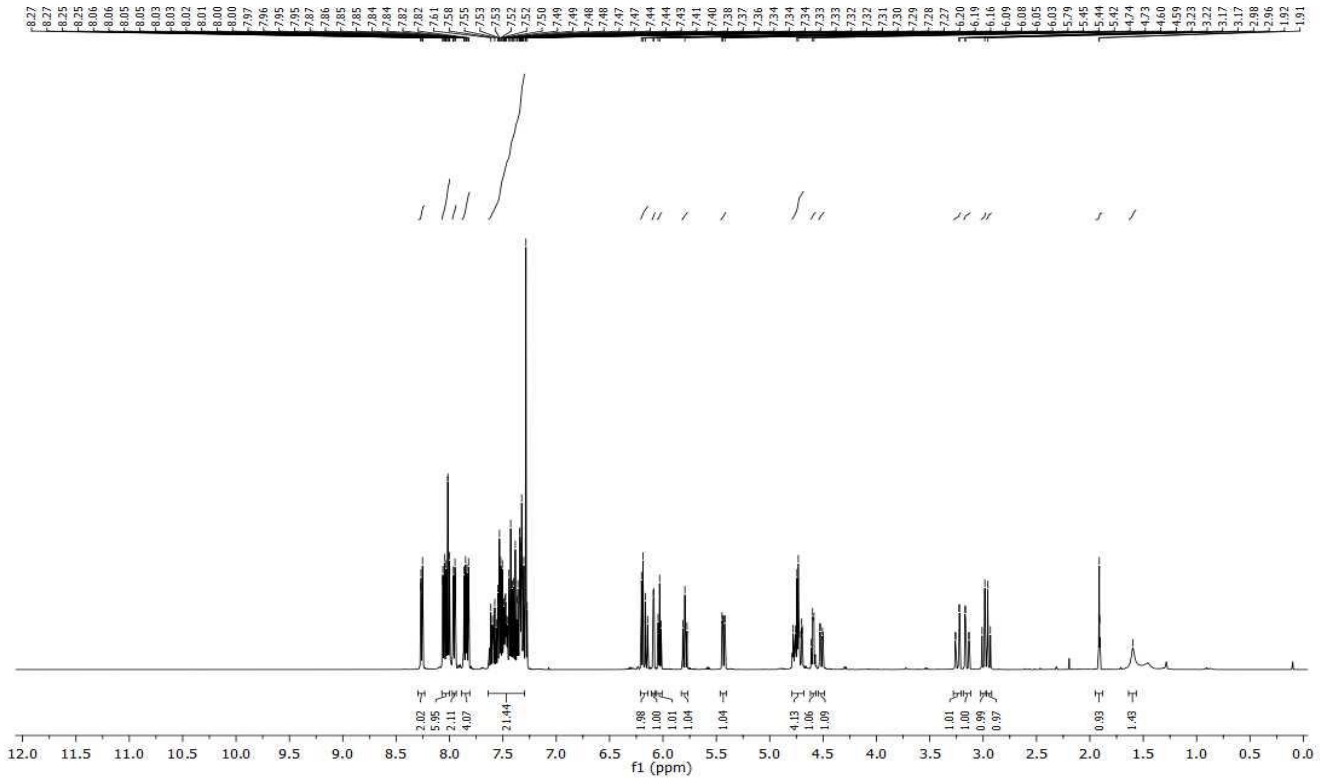

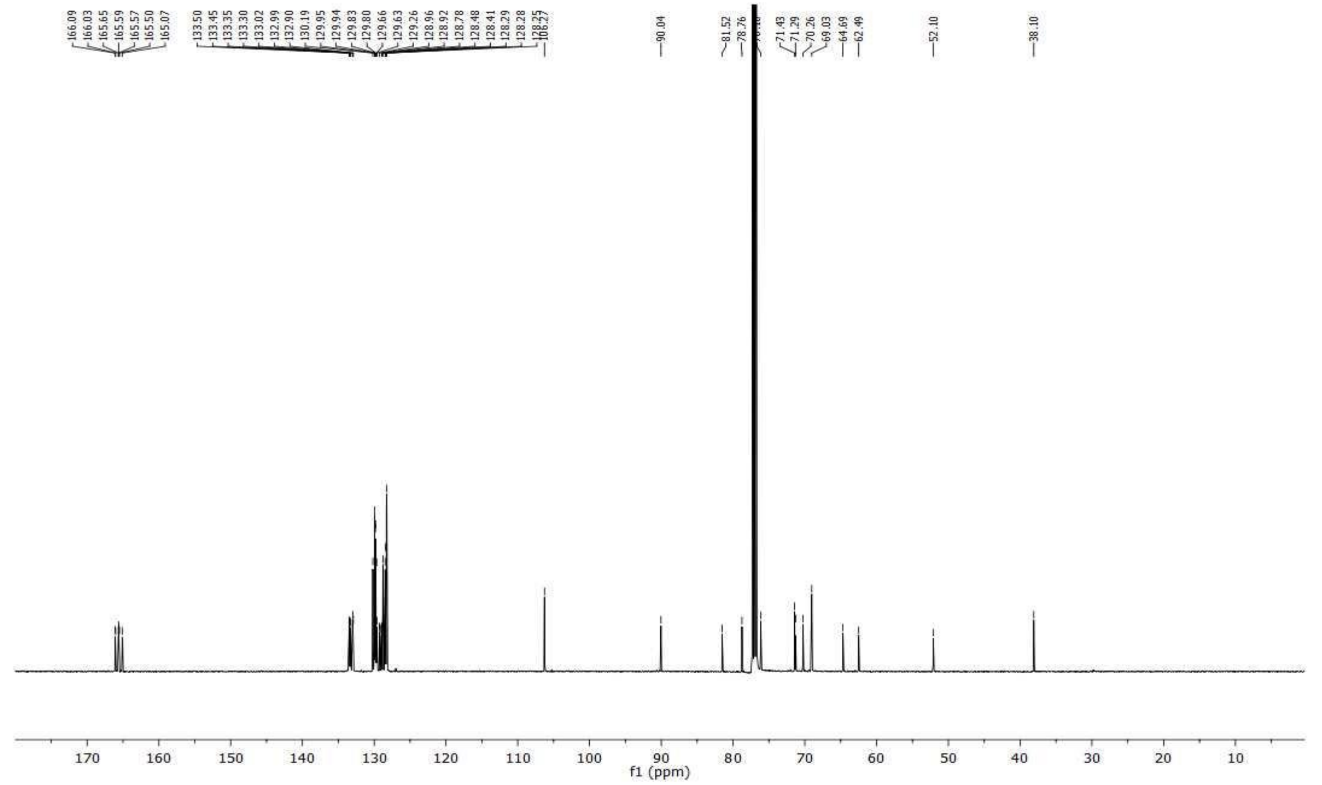

Compound **6**

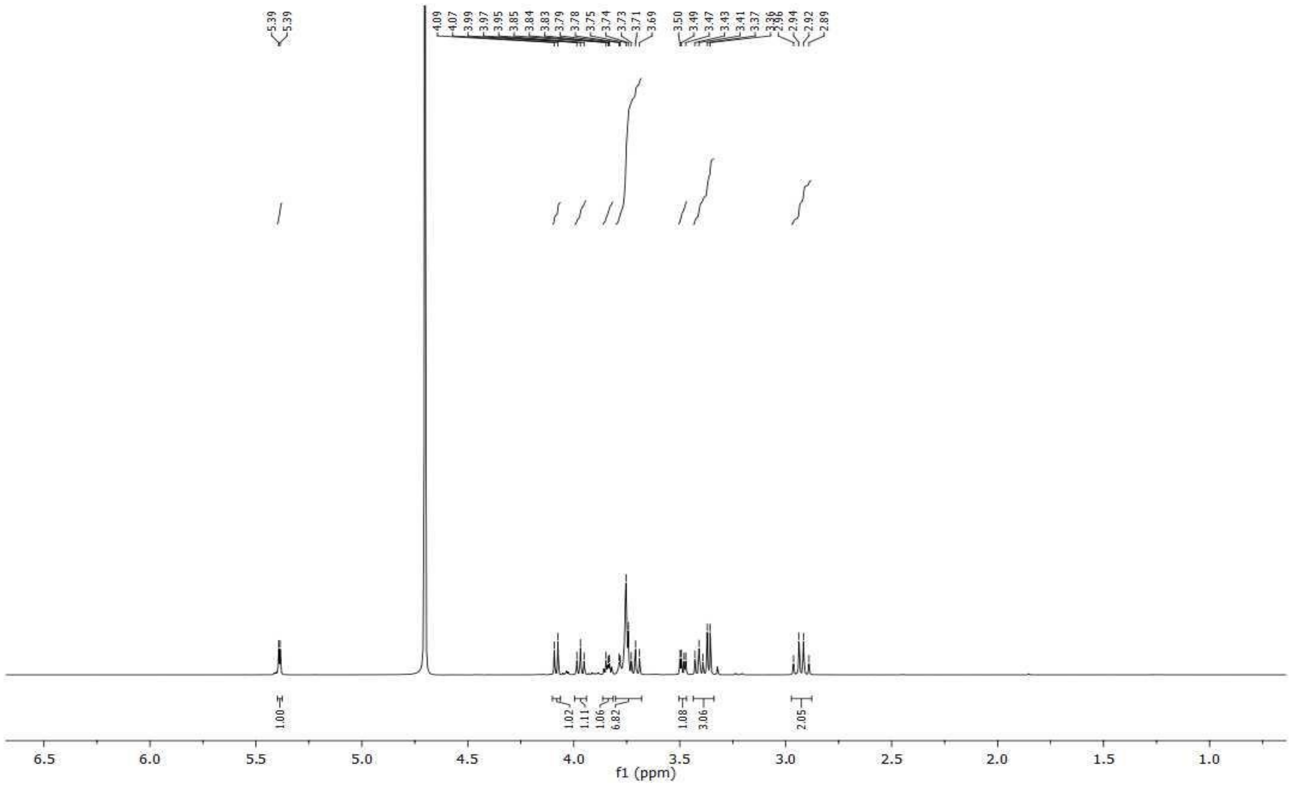

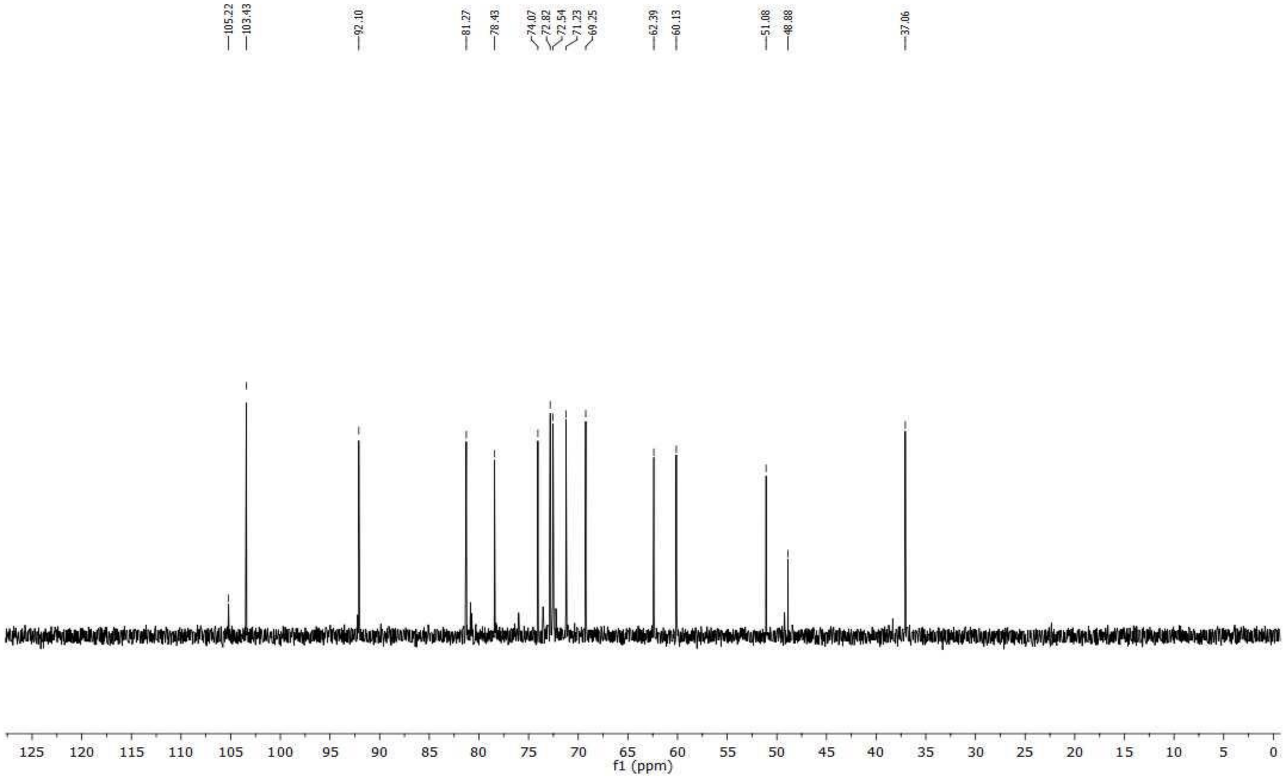

Compound **8** (contains traces of residual EtOAc)

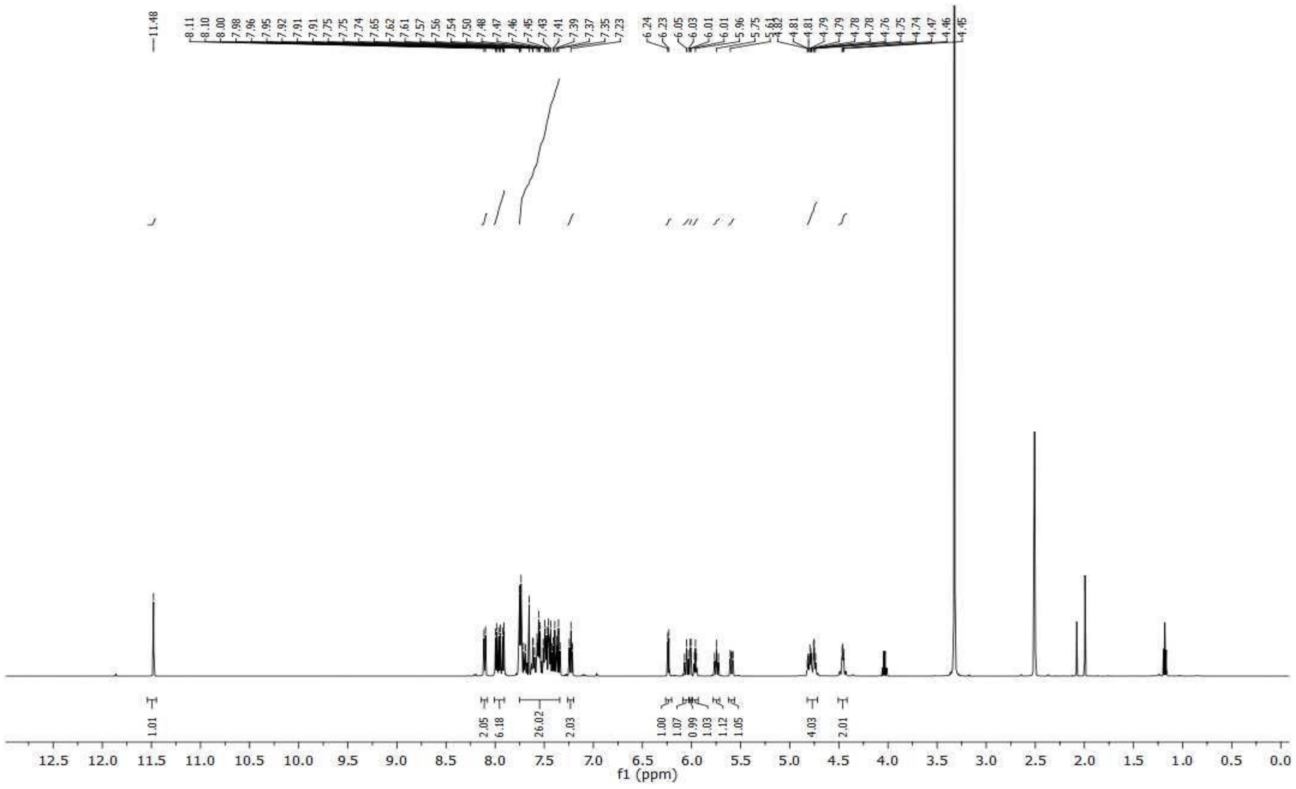

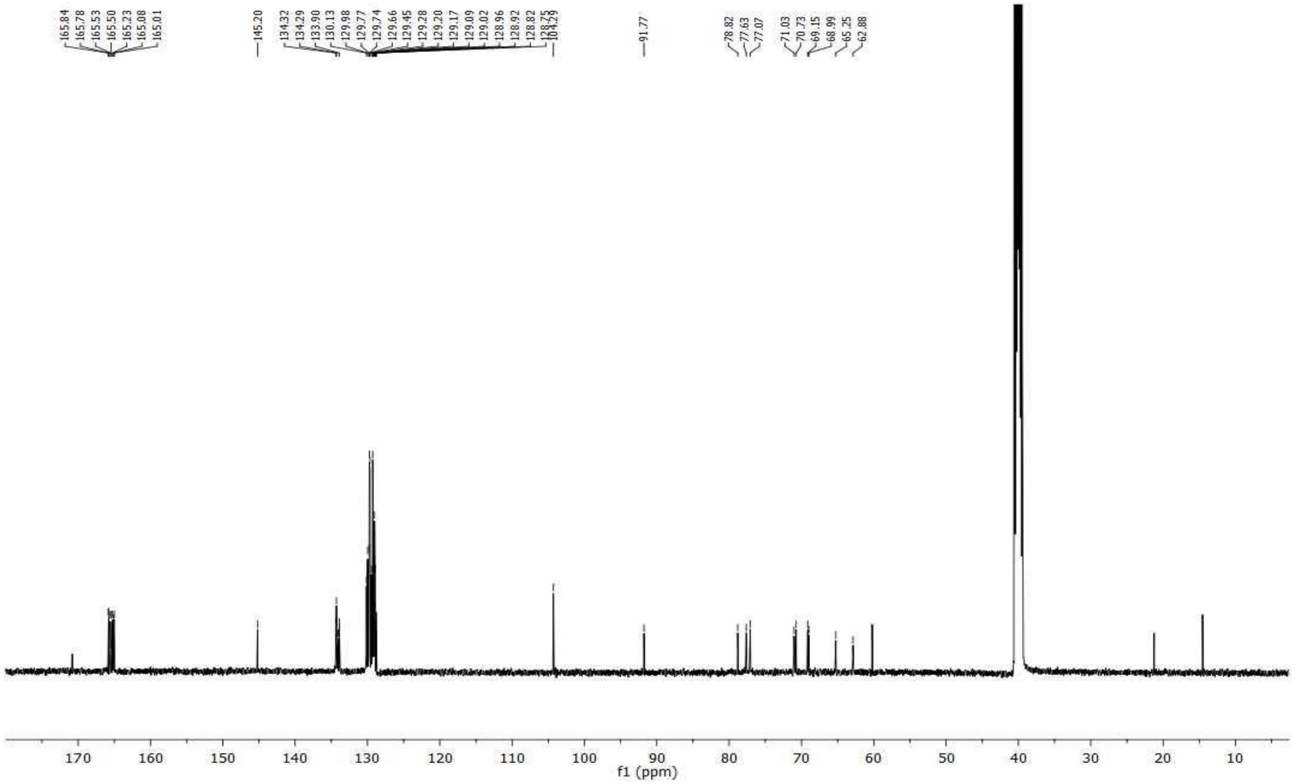

Compound **9**

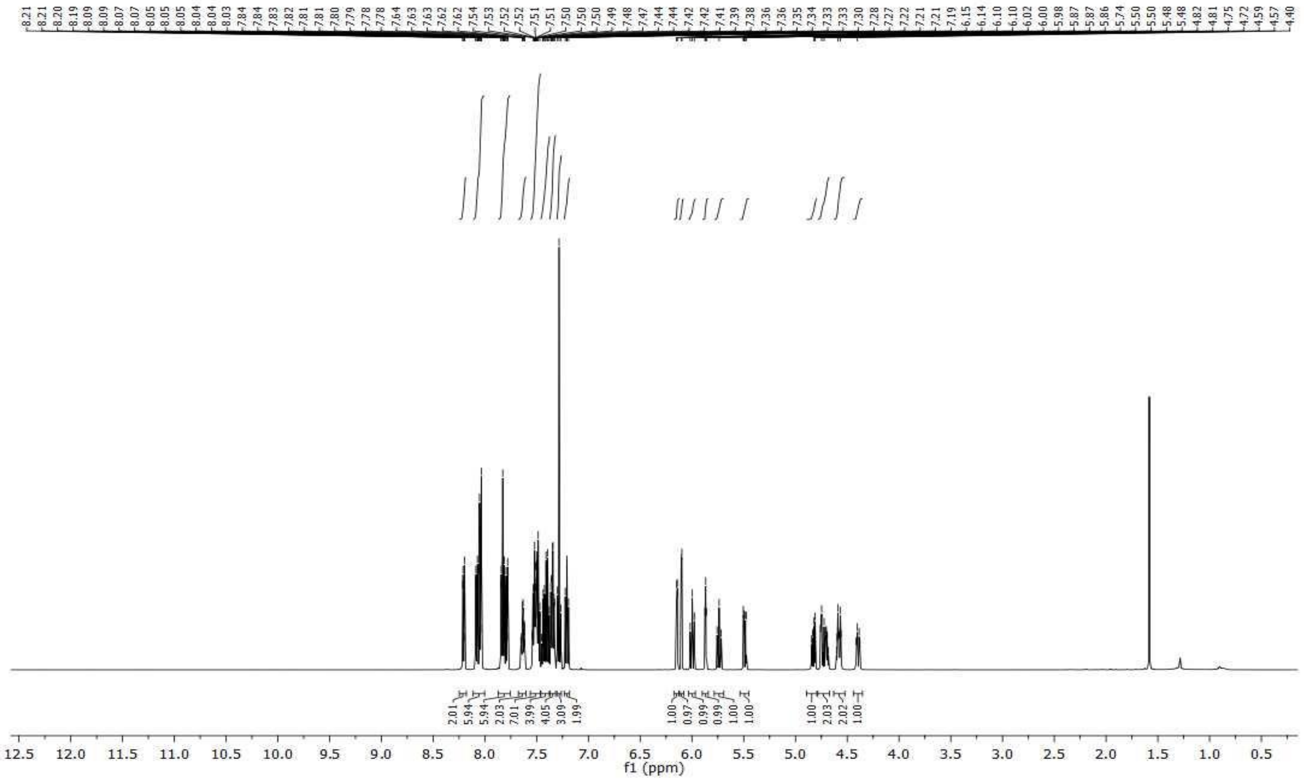

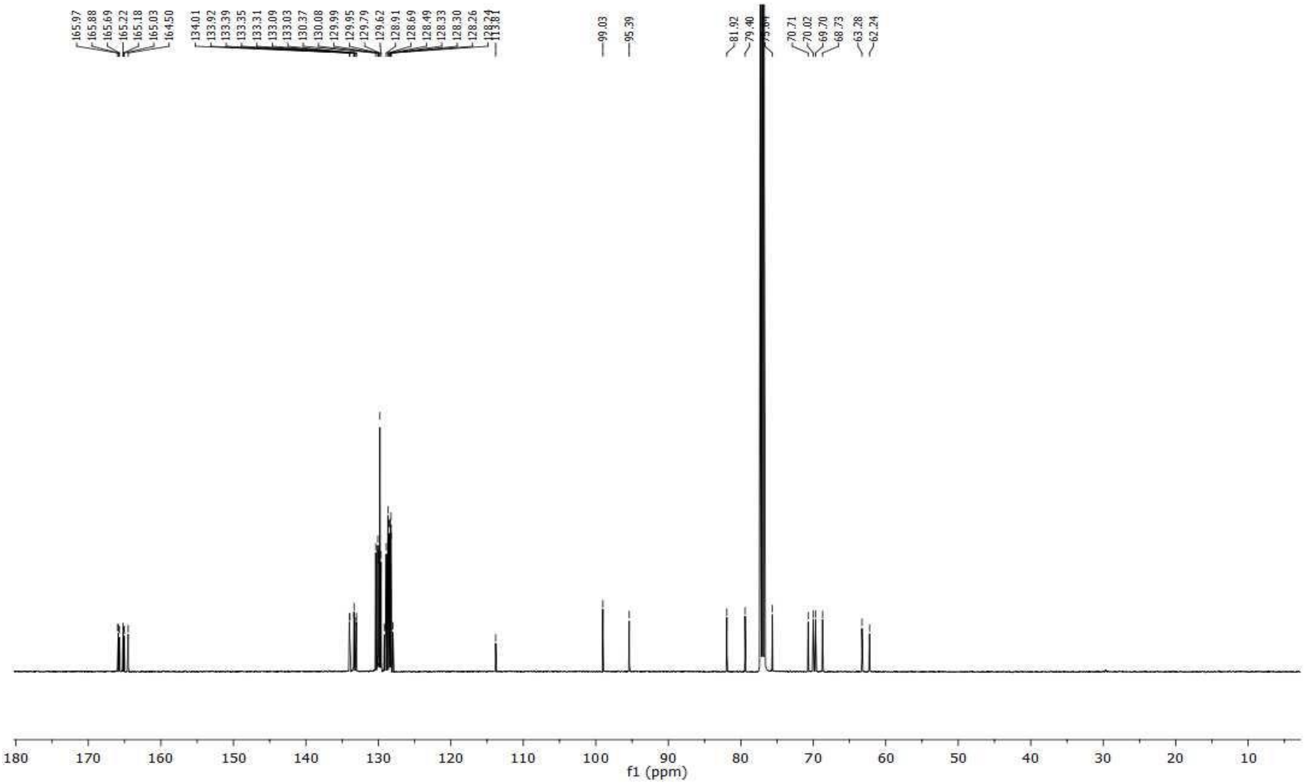

Compound **11**

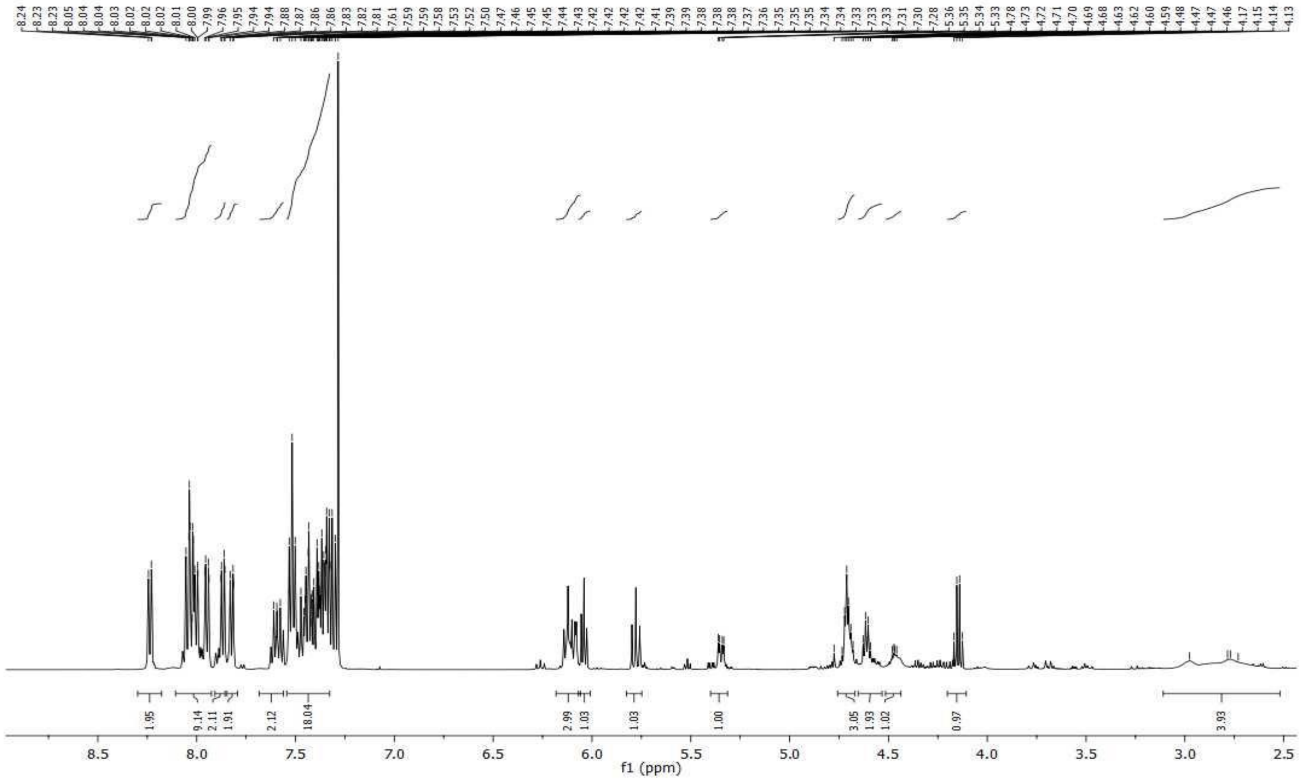

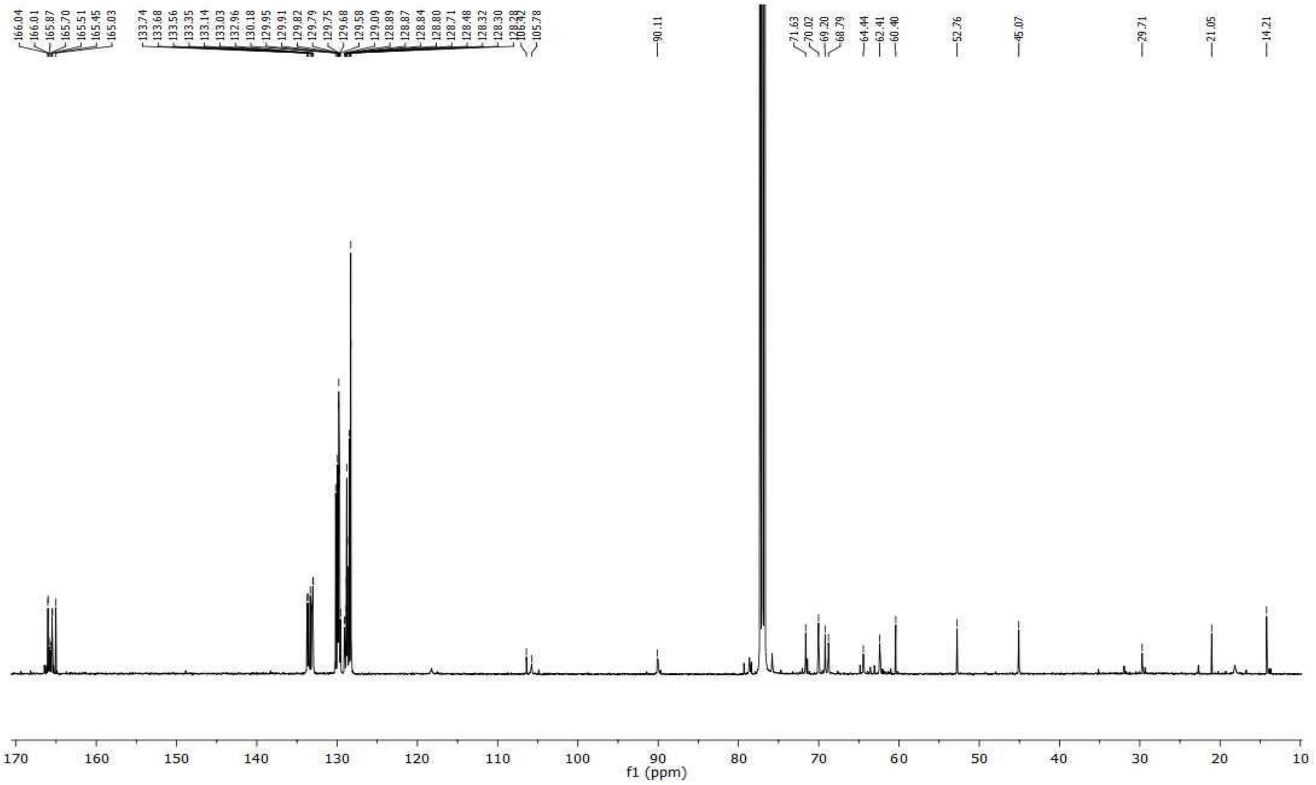

Compound **12**

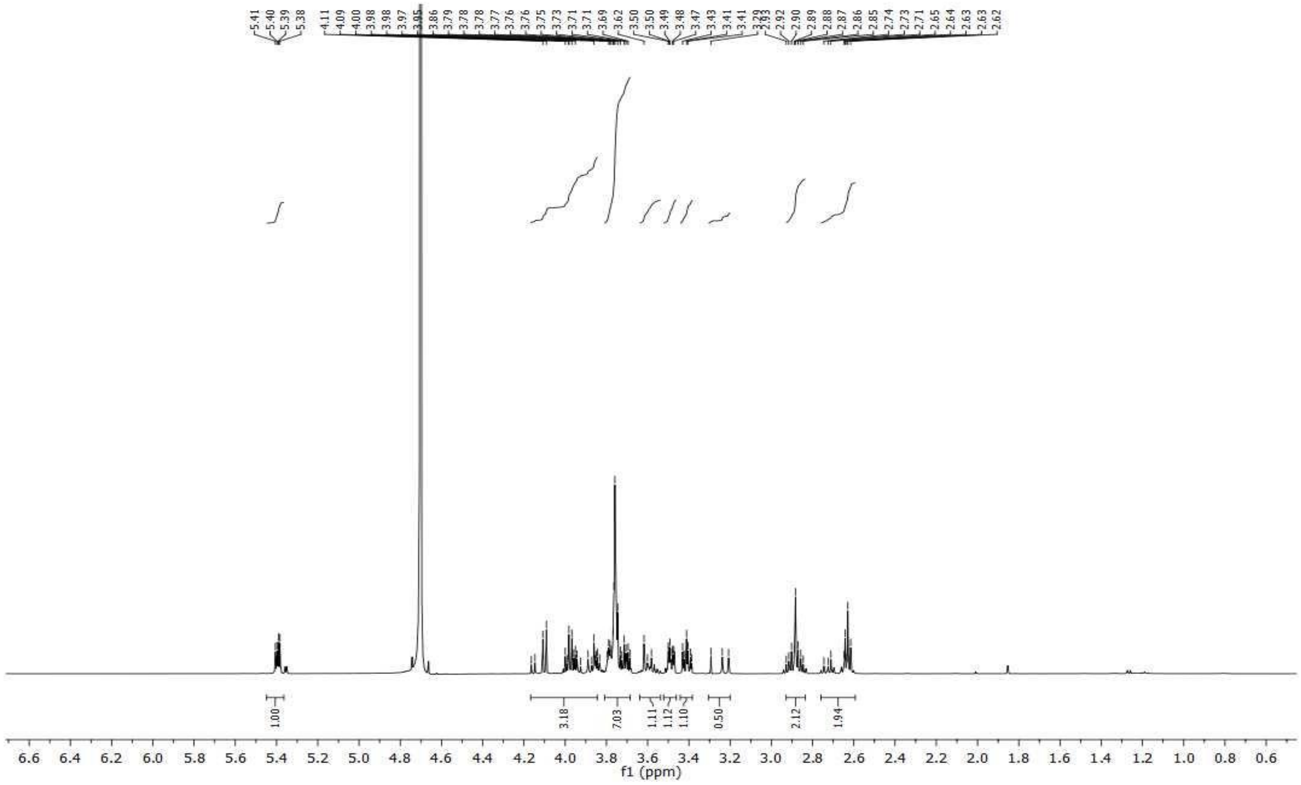

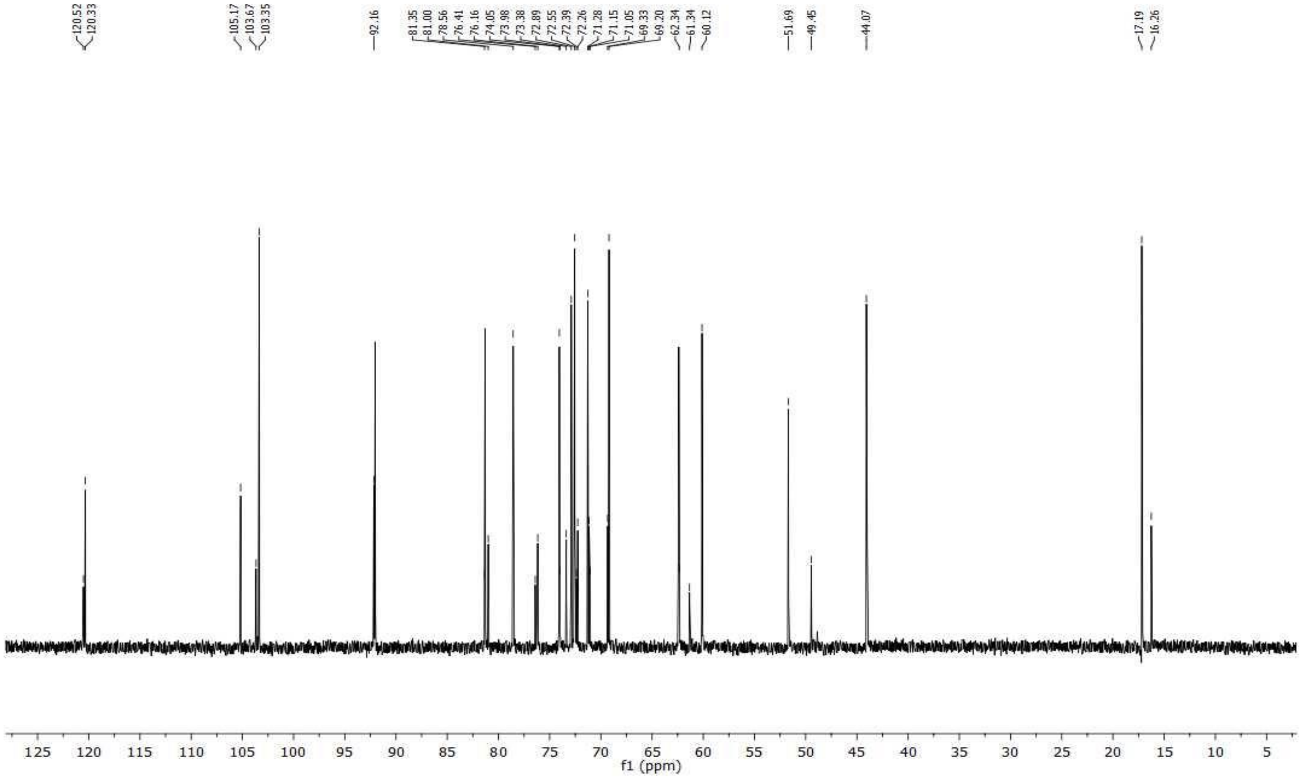

3. Supplementary Methods (Spectroscopy/Biology/Imaging)

Raman spectroscopy. Spontaneous Raman spectra were acquired using a confocal Raman spectrometer (inVia Raman microscope, Renishaw) at r.t. using laser excitation sources at 532 nm (50 mW) or 785 nm (297 mW) through a 50× objective lens (NA: 0.75). Raman spectra were processed in WiRE 4.1™ software. Raman spectra of compounds **4**, **6**, **12** and **13** were acquired at 785 nm under 50% laser power (~95 mW). The limits of detection for compounds **6** and **13** were determined in water containing DMSO (5% v/v as the internal standard) at 785 nm using 100% laser power (~180 mW). Cosmic rays were removed, spectra were smoothed using Savitzky-Golay filtering with a polynomial order of 3 and a frame length of 9 using the in-built functions available on WiRE 4.1 and baseline corrected. The Raman spectra were normalised to the intensity of the peak at 2927 cm^{-1} (DMSO) and the peak areas (2085-2160 cm^{-1} for compound **6**, 2075-2155 cm^{-1} for compound **13**) were determined using the integrate tool available on OriginPro 2018 software. Raman spectra of BY2 cell pellets were acquired at 532 nm under 100% laser power (~30 mW). The spectrum was baseline-corrected using WiRE 4.1.

***In vitro* uptake assays in yeast cells.** The uptake of compound **6** was examined in the SEY6210 yeast strain transformed with either pDR196 (vector control) or sucrose transporters StSUT1 and AtSUC2 in pDR196, or OsSUT1 in pDR196/GW, as described previously.[2] Yeast transformed with the empty vector pDR196 was used as the negative control. Briefly, colonies of each yeast cell line were inoculated in synthetic defined media lacking uracil (SD-URA) supplemented with 2% glucose and grown for 24 h at 30 °C with shaking at 300 rpm.[3] Cell cultures were diluted to an OD_{600} of 0.4 in fresh SD-URA media and further incubated for 3 h until they reached an OD_{600} of 0.6-0.8. Then, cell cultures

were centrifuged at 500 *g* and washed once with 25 mM NaH_2PO_4 buffer to obtain cell pellets. 5×10^5 cells from each yeast line were resuspended and incubated with compound **6** in buffer for 2 h at 30 ºC with shaking. Cells were harvested and washed once before being incubated for 20 min at 30 ºC with AHC (1 mM, 1% DMSO), THPTA (1 mM in 25 mM NaH_2PO_4), $CuSO_4$ (10 mM in 25 mM NaH_2PO_4) and sodium ascorbate (10 mM in 25 mM NaH_2PO_4) in a final volume of 300 μL. Cell suspensions were then vortexed and plated in black 96-well plates to measure their fluorescence emission in a Biotek spectrophotometer (λ_{exc}: 400 nm, λ_{em}: 460 nm).

***In vitro* sucrose invertase assays**. Compound **6** or sucrose were dissolved in miliQ water at the indicated concentrations and incubated with sucrose invertase (150 U mL^{-1}) for 10 min at r.t. Afterwards, the mixtures were further incubated for 15 min at r.t. with a cocktail of glucose hexokinase (1 U mL^{-1}), 1 mM ATP, glucose-6-phosphate dehydrogenase (1 U mL^{-1}) and 1.5 mM NAD. Finally, absorbance readings were taken at 340 nm (i.e., directly proportional to the concentration of NADH) in a Biotek spectrophotometer. Data was processed with GraphPad Prism 8.0.

Functional Ca^{2+} influx assays. The SEY6210 yeast strain transformed with StSUT1 transporters was used for these assays. 10^6 cells were incubated with compound **6** (100 mM) in 25 mM NaH_2PO_4 buffer (containing 1 mM $CaCl_2$ or not) for 2 h at 30 ºC with shaking and then further treated with Fluo-4 AM (5 μM) and incubated for additional 60 min at 30 ºC. Cells were then washed once, resuspended in buffer (300 μL) and their fluorescence emission was measured in a Biotek spectrophotometer (λ_{exc}: 490 nm, λ_{em}: 510 nm).

Culture of BY2 plant cells. Tobacco (*Nicotiana tabacum*) BY2 cells were cultured aseptically in 250 mL Erlenmeyer flasks containing 80 mL of sterile 4.3 g L^{-1} Murashige and Skoog (MS) basal salt medium, 30 g L^{-1} sucrose and 200 µg mL^{-1} of 2,4-dichlorophenoxyacetic acid sodium salt monohydrate at 28 °C in the dark with continuous shaking at 140 rpm. Cells were sub-cultured with fresh medium every 7 days. For sucrose starvation, cells were cultured for 18 h prior to imaging, in media where sucrose was replaced by mannitol. For imaging, 10 µL of cells were incubated with 10 µL of compound **6** at the indicated concentrations in BY2 media (without sucrose), applied to a microscope slide, sealed with a coverslip, and imaged immediately.

Live-cell microscopy and image analysis. Briefly, a picoEmerald (APE) laser provided both a tunable pump laser (720–990 nm, 7 ps, 80 MHz repetition rate) and a spatially overlapped second beam termed the Stokes laser (1064 nm, 5–6 ps and 80 MHz repetition rate). The laser was inserted into an Olympus FV1000MPE microscope coupled to an Olympus XLPL25XWMP N.A. 1.05 objective lens using a short-pass 690 nm dichroic mirror (Olympus). For SRS measurements, the Stokes beam was modulated with a 20 MHz EoM. Forward scattered light was collected by a 20× Olympus XLUMPLFLN Objective, 1.00 NA lens and filtered using ET890/220m filter (Chroma). A telescope focused the light onto an APE silicon photodiode connected to an APE lock in amplifier which was fed into the analogue unit of the microscope.[4] The pump laser was tuned to match the vibrational shift being measured. For alkyne images a corresponding off-resonance images were taken at ± 25 cm^{-1} and subtracted from the on-resonance images. Laser powers after the objective were measured at 40-70 mW for the pump laser and 60 mW for the Stokes laser. All images were recorded at 512 × 512 or 1024 × 1024 pixels

with a pixel dwell time between 2 and 20 μs by FV10-ASW software (Olympus). Image analysis and processing were performed in ImageJ.

4. Supplementary Figures

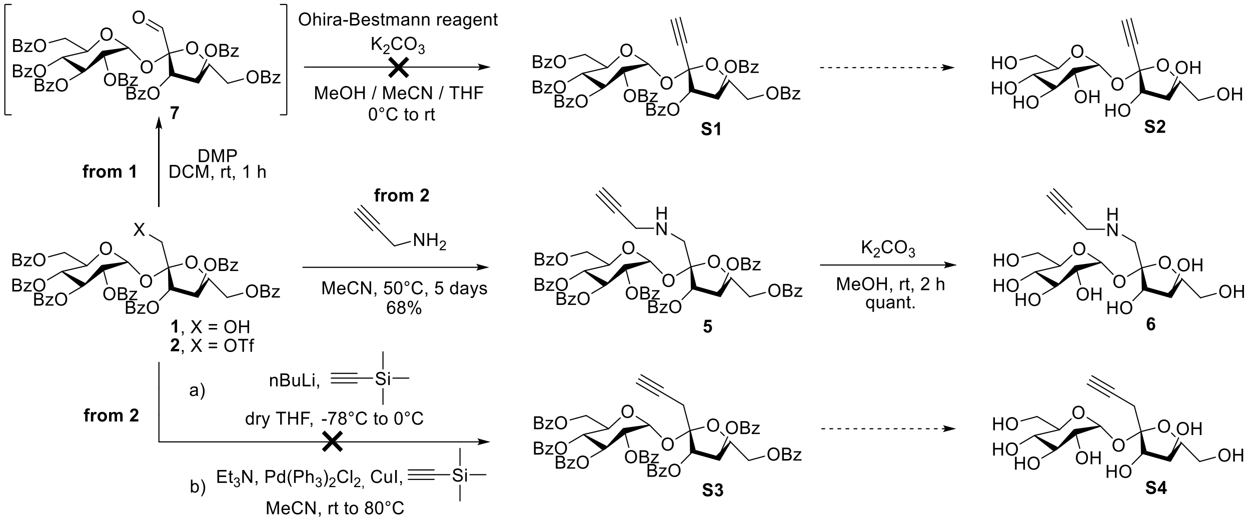

Supplementary Figure 1. Different synthetic strategies considered the preparation of an alkyne-endowed Raman active sucrose analogues. Installation of an alkyne moiety by means of a reaction between sucrose triflate **2** and trimethylsilylacetylene to give **S3** (bottom) failed under both strong basic conditions and Sonogashira coupling conditions, leaving starting material **2** mostly unreacted. A functional group interconversion approach to convert an aldehyde group into a triple bond (top) to give **S1** resulted in decomposition of intermediate **7**.

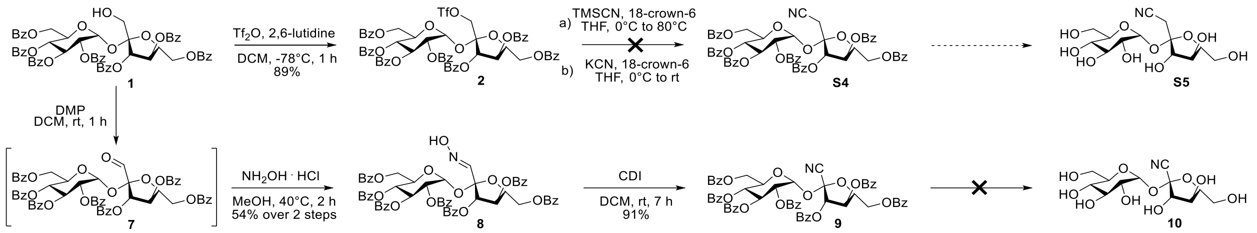

Supplementary Figure 2. **Different synthetic strategies considered the preparation of nitrile-containing Raman active sucrose analogues**. Nucleophilic substitution exploiting either KCN or TMSCN (top) failed to convert sucrose triflate **2** into nitrile **S4** and resulted in extensive decomposition of the starting material. Generation of nitrile intermediate **9** through dehydration of a sucrose oxime **8** employing CDI worked smoothly under mild conditions (bottom), but the final deprotection step to afford sucrose nitrile **10** proved unfeasible (See Supplementary Table 1).

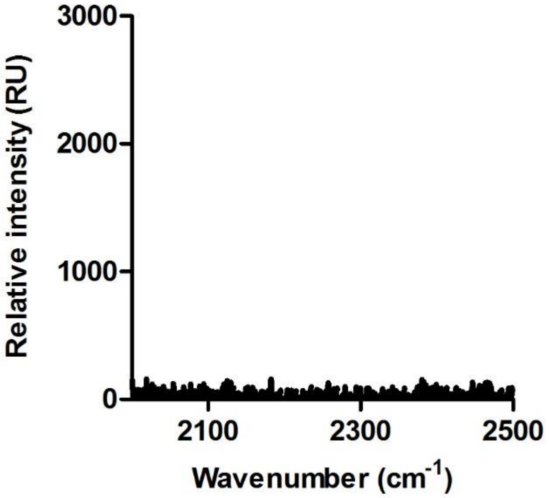

Supplementary Figure 3. Raman spectrum of native sucrose. Representative Raman spectrum of solid sucrose covering the silent biological window (n=3).

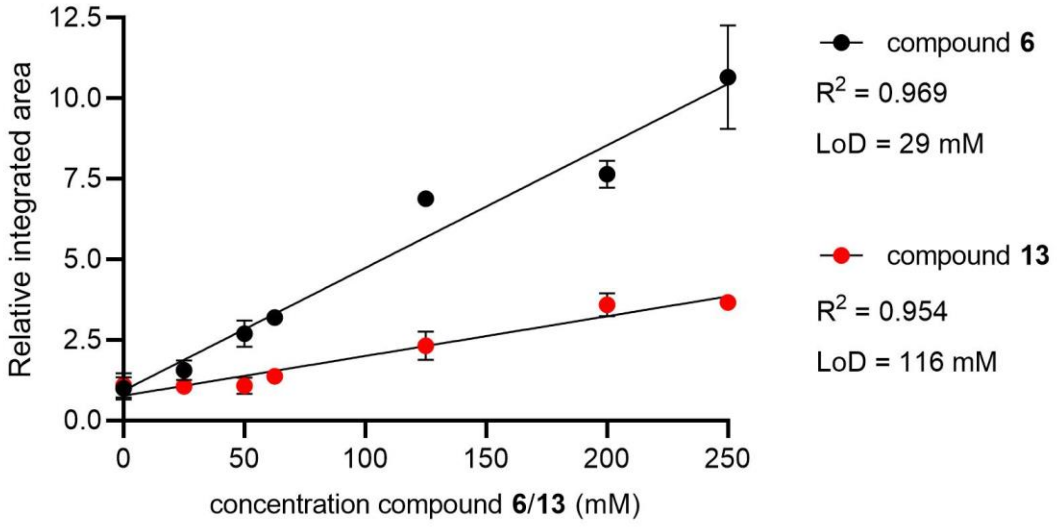

Supplementary Figure 4. Limits of detection for compounds 6 and 13. Compounds were dissolved in water at the indicated concentrations and relative integrated areas for the Raman peaks (2085-2160 cm^{-1} for compound **6**, 2075-2155 cm^{-1} for compound **13**) were determined in a confocal Raman spectrometer (inVia Raman microscope, Renishaw). The limits of detection were determined as means of the blank plus 3×standard deviation of the blank. Data presented as means±SD (n=3).

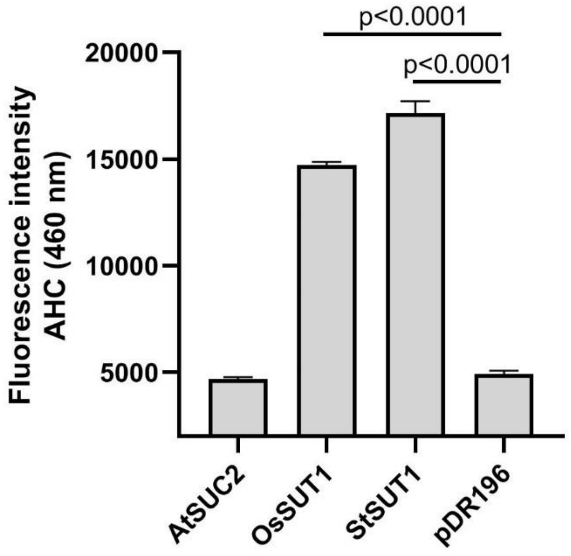

Supplementary Figure 5. Uptake of compound 6 in genetically-modified yeast cells. Fluorescence intensity (λ_{exc}: 400 nm, λ_{em}: 460 nm) of cell pellets from yeast mutants expressing SUT1 and SUC2 sucrose transporters or the vector control (pDR196) after incubation with compound **6** (100 mM) and subsequent reaction 3-azido-7-hydroxycoumarin (AHC, 1 mM), THPTA (1 mM), $CuSO_4$ (10 mM) and sodium ascorbate (10 mM). Data presented as means±SD (n=3) with p values obtained from one-way ANOVA with multiple comparisons.

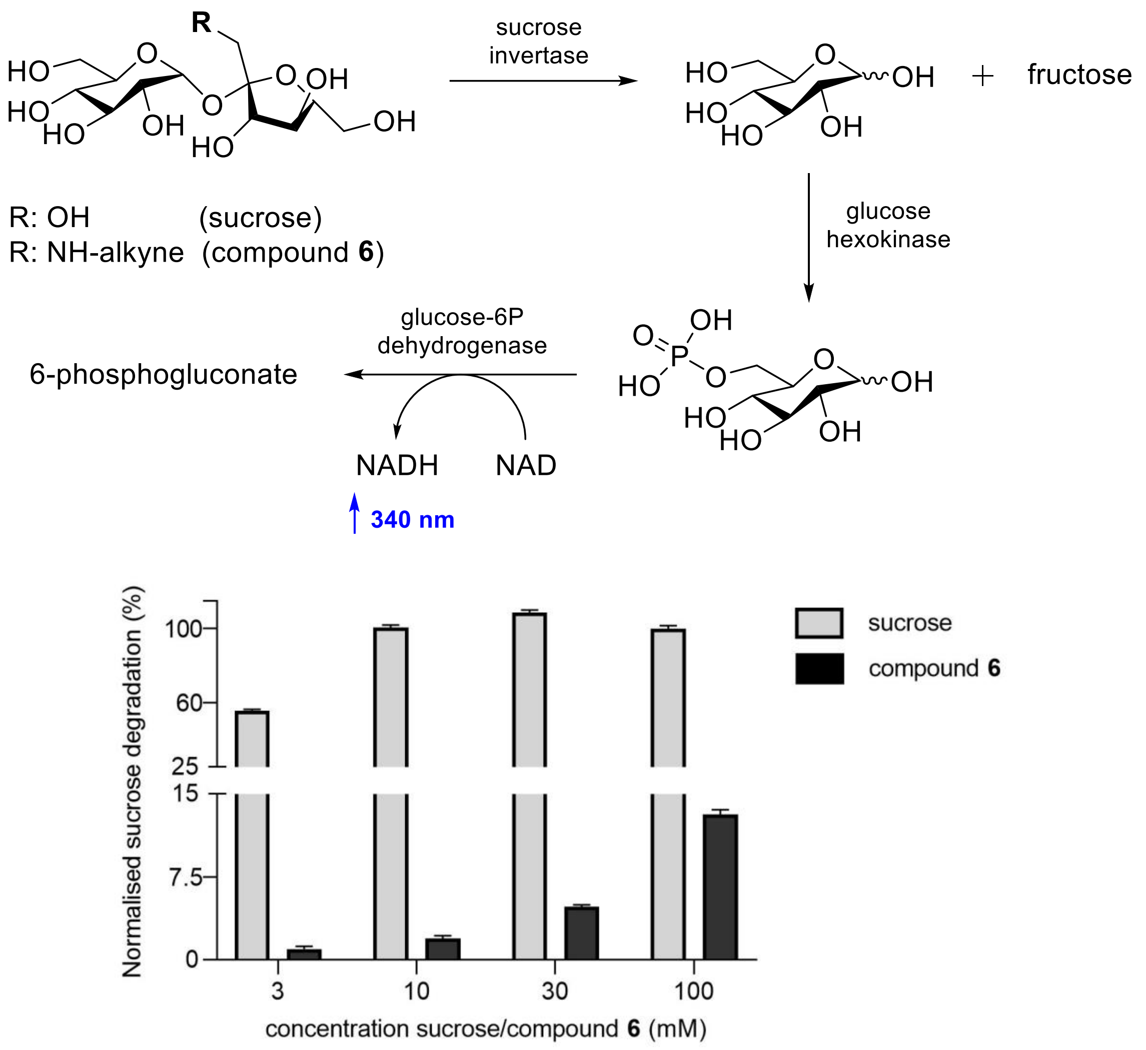

Supplementary Figure 6. Reactivity of sucrose and compound 6 against sucrose invertase. Compound **6** or sucrose were dissolved in miliQ water at the indicated concentrations and incubated with sucrose invertase (150 U mL^{-1}) for 10 min at r.t. Mixtures were further incubated for 15 min at r.t. with a cocktail of glucose hexokinase (1 U mL^{-1}), 1 mM ATP, glucose-6-phosphate dehydrogenase (1 U mL^{-1}) and 1.5 mM NAD. Absorbance readings were taken at 340 nm (concentration of NADH) and data was processed with GraphPad Prism 8.0. Data presented as means±SD (n=6).

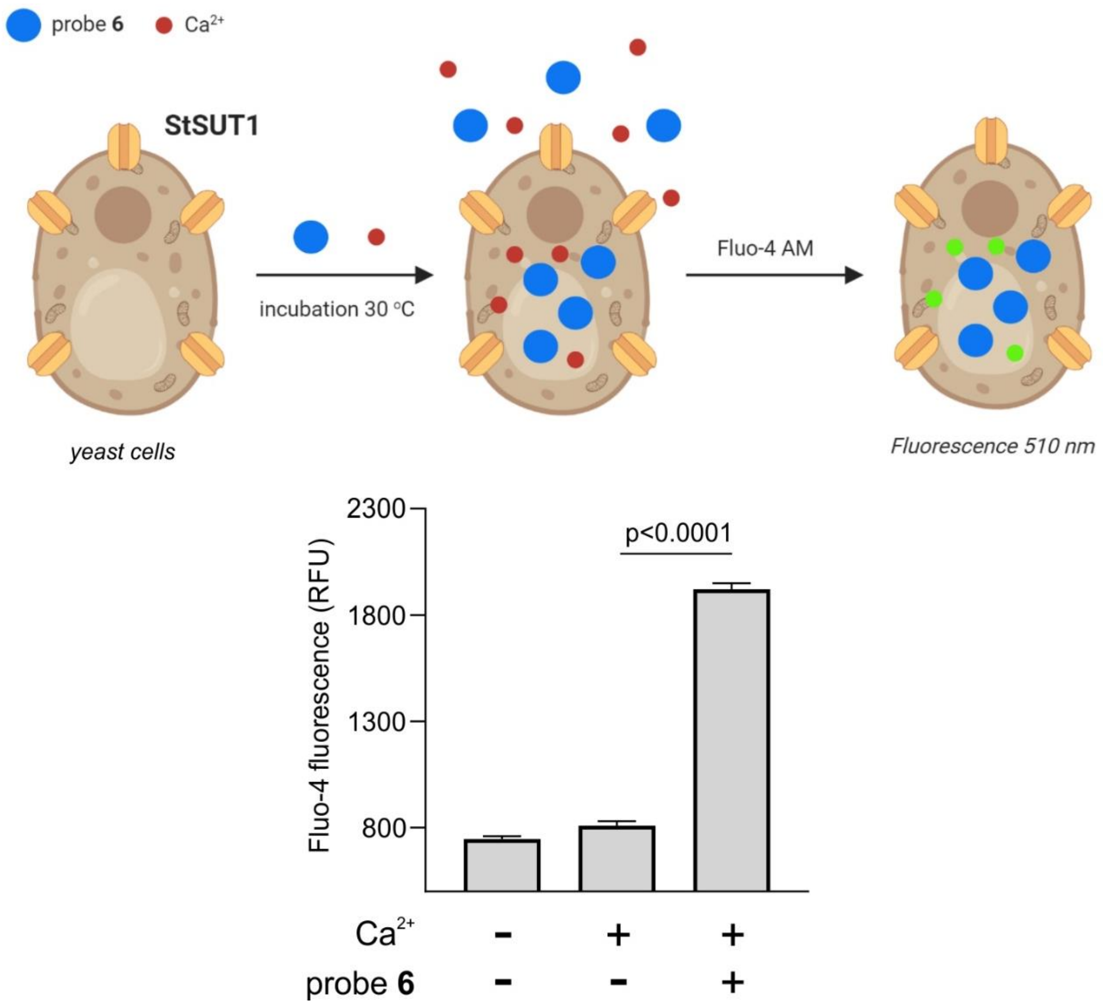

Supplementary Figure 7. Functional Ca^{2+} influx assays. StSUT1-expressing yeast cells (10^6 cells/test) were incubated with compound **6** (100 mM) in 25 mM NaH_2PO_4 buffer (containing 1 mM $CaCl_2$ or not) for 2 h at 30 ºC and then further treated with Fluo-4 AM (5 μM) for 60 min at 30 ºC. Cells were washed and resuspended in buffer to measure their fluorescence emission (λ_{exc}: 490 nm, λ_{em}: 510 nm). Data presented as means±SD (n=3) with p values obtained from unpaired *t* tests.

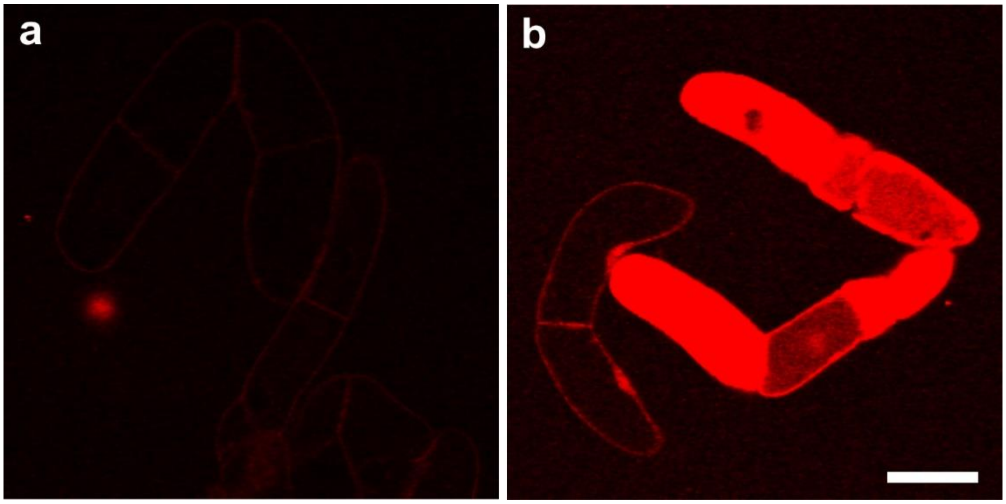

Supplementary Figure 8. Analysis of cytotoxicity of compound 6 in BY2 cells. A) Representative fluorescence microscope images (from 3 independent experiments) of BY2 cells upon incubation with compound **6** (125 mM) and propidium iodide as a marker of dead cells. B) Positive control: propidium iodide-stained dead BY2 cells. Scale bar: 50 μm.

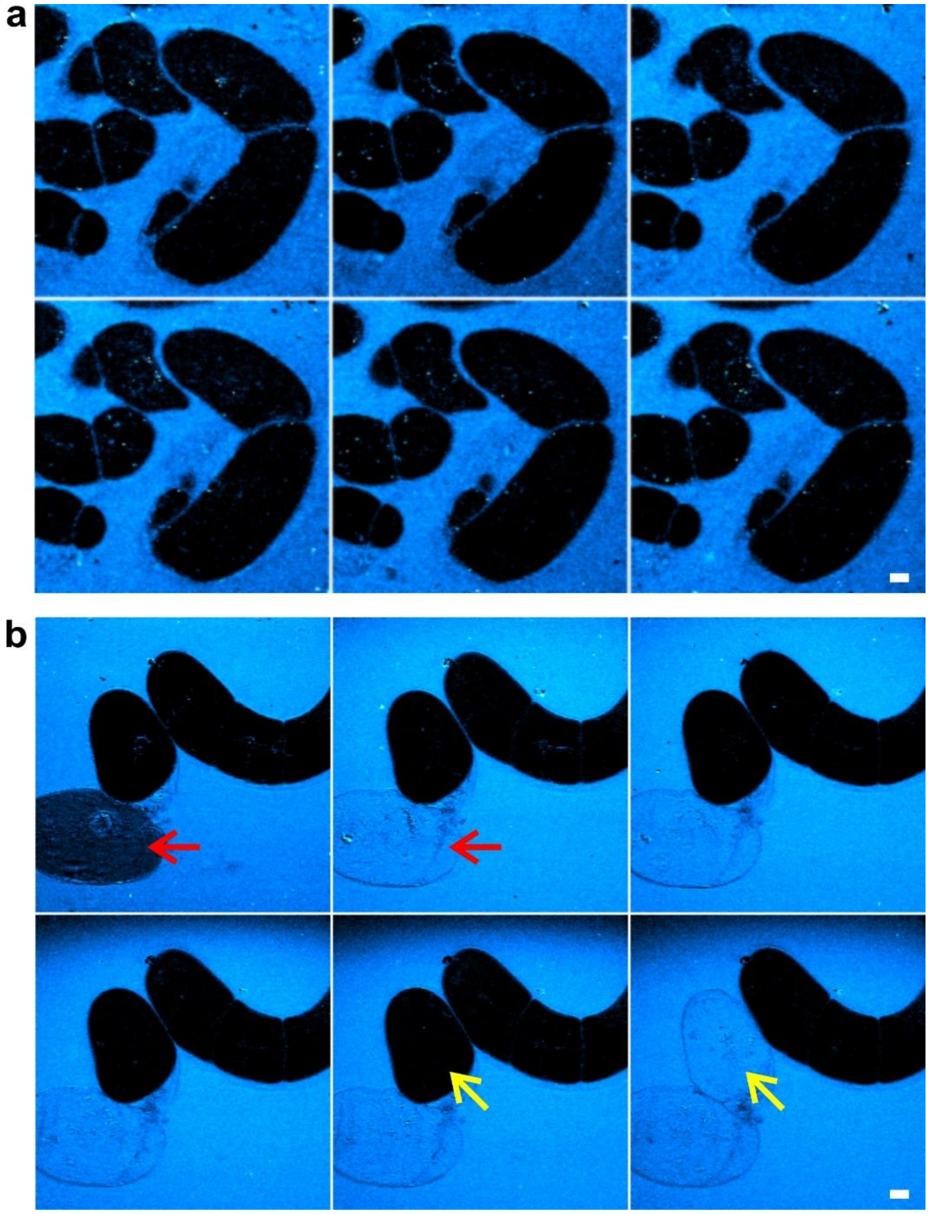

Supplementary Figure 9. SRS microscope images show competition between sucrose and compound 6 for intracellular accumulation in vacuoles. a) Time-lapse pseudo-colour Raman images (snapshots every 3 sec, wavenumber: 2,116 cm^{-1}) of BY2 cells after co-incubation of sucrose and compound **6** (both at 125 mM) where compound **6** does not show intracellular accumulation (full recording in Supplementary Movie 1). b) Time-lapse pseudo-colour Raman images (snapshots every 3 sec, wavenumber: 2,116 cm^{-1}) of BY2 cells after incubation with compound **6** only (125 mM). Arrows highlight

intracellular vacuolar accumulation in different cells (red: cell 1; yellow: cell 2). Full recording in Supplementary Movie 2. Scale bars: 10 μm.

Supplementary Movie 1. Recording of BY2 cells after co-incubation of sucrose and compound 6. Longitudinal Raman imaging was acquired at 2,116 cm^{-1} (10 frames per second) and processed with ImageJ.

Supplementary Movie 2. Recording of BY2 cells after incubation with compound 6 alone. Longitudinal Raman imaging was acquired at 2,116 cm^{-1} (10 frames per second) and processed with ImageJ.

5. Supplementary Tables

Supplementary Table 1. Reaction conditions screened for the deprotection of the perbenzoylated precursor **9** to afford the nitrile-endowed Raman active sucrose analogue **10**.

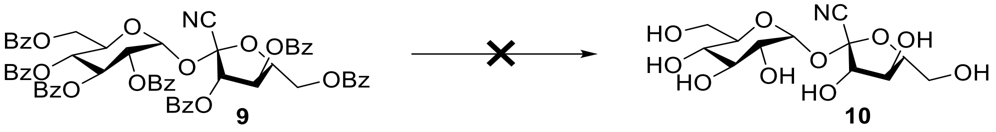

Entry	Base / Acid	Temperature	Solvent	Reaction Time	Outcome
1	K_2CO_3	rt	THF	Overnight	No reaction
2	K_2CO_3	reflux	THF	Overnight	No reaction
3	K_2CO_3	rt	MeOH	Overnight	Mixture of partially deprotected products
4	K_2CO_3	reflux	MeOH	1 h	Decomposition
5	K_2CO_3	rt	iPrOH	Overnight	Mixture of partially deprotected products
6	K_2CO_3	80°C	iPrOH	1 h	Nucleophilic addition of the solvent
7	K_2CO_3	30°C	tBuOH	Overnight	No reaction
8	K_2CO_3	80°C	tBuOH	Overnight	No reaction
9	K_2CO_3	rt	TFE	1 h	Mixture of partially deprotected products
10	K_2CO_3	rt	TFE	6 h	Nucleophilic addition of the solvent
11	K_2CO_3	rt	Me_2CO	1 h	Mixture of partially deprotected products
12	K_2CO_3	rt	Me_2CO	6 h	Hydrolysis to potassium carboxylate
13	K_2CO_3	rt	DMF	Overnight	Mixture of partially deprotected products
14	K_2CO_3	50°C	DMF	2 h	Hydrolysis to potassium carboxylate
15	K_2CO_3	rt	H_2O	2 h	Hydrolysis to potassium carboxylate
16	$CsCO_3$	rt	THF	Overnight	No reaction
17	$CsCO_3$	reflux	THF	Overnight	Mixture of partially deprotected products
18	$CsCO_3$	100°C	Dioxane	2 h	Decomposition
19	NaOH	rt	THF	30 min	Decomposition
20	NaOH	rt	MeOH	30 min	Decomposition
21	Et_3N	rt	THF	Overnight	No reaction
22	Et_3N	reflux	THF	Overnight	No reaction
23	Et_3N	rt	MeOH	Overnight	No reaction
24	Et_3N	reflux	MeOH	Overnight	No reaction
25	HCl	rt	THF	30 min	Decomposition
26	HCl	rt	MeOH	30 min	Decomposition

6. Supplementary References

1. W. Ying, V. Gaddam, M. Harmata, *Org. Lett.* **2013**, *15*, 2723-2735.
2. P. J. Gora, A. Reinders, J. M. Ward. *Plant Methods* **2012**, *8*, 13.
3. L. F. Pemberton, in *Yeast Genetics: Methods and Protocols* (Eds.: J.S. Smith, D.J. Burke), Springer, New York, **2014**, pp. 79–90.
4. W. J Tipping, M. Lee, A. Serrels, V. G. Brunton, A. N. Hulme, *Chem. Sci.* **2017**, *8*, 5606-5615.
